# Mycoplasma glycine cleavage system key subunit GcvH is an apoptosis inhibitor targeting host endoplasmic reticulum

**DOI:** 10.1371/journal.ppat.1012266

**Published:** 2024-05-24

**Authors:** Qiao Pan, Yujuan Zhang, Tong Liu, Qingyuan Xu, Qi Wu, Jiuqing Xin

**Affiliations:** 1 State Key Laboratory for Animal Disease Control and Prevention, Harbin Veterinary Research Institute, Chinese Academy of Agricultural Sciences, Harbin, China; 2 Institute of Western Agriculture, Chinese Academy of Agricultural Sciences, Xinjiang, China; Miami University, UNITED STATES

## Abstract

Mycoplasmas are minimal but notorious bacteria that infect humans and animals. These genome-reduced organisms have evolved strategies to overcome host apoptotic defense and establish persistent infection. Here, using *Mycoplasma bovis* as a model, we demonstrate that mycoplasma glycine cleavage system (GCS) H protein (GcvH) targets the endoplasmic reticulum (ER) to hijack host apoptosis facilitating bacterial infection. Mechanically, GcvH interacts with the ER-resident kinase Brsk2 and stabilizes it by blocking its autophagic degradation. Brsk2 subsequently disturbs unfolded protein response (UPR) signaling, thereby inhibiting the key apoptotic molecule CHOP expression and ER-mediated intrinsic apoptotic pathway. CHOP mediates a cross-talk between ER- and mitochondria-mediated intrinsic apoptosis. The GcvH N-terminal amino acid 31–35 region is necessary for GcvH interaction with Brsk2, as well as for GcvH to exert anti-apoptotic and potentially pro-infective functions. Notably, targeting Brsk2 to dampen apoptosis may be a conserved strategy for GCS-containing mycoplasmas. Our study reveals a novel role for the conserved metabolic route protein GcvH in *Mycoplasma* species. It also sheds light on how genome-reduced bacteria exploit a limited number of genomic proteins to resist host cell apoptosis thereby facilitating pathogenesis.

## Author summary

Mycoplasma-related debilitating and chronic diseases have long troubled human and animal health. A better understanding of mycoplasma pathogenesis helps to develop effective control strategies. Some mycoplasmas have been reported to delay or inhibit host apoptosis, which could be an important pathogenic mechanism for *Mycoplasma* organisms. In the present study, we propose the first molecular mechanism model describing how GCS-containing mycoplasmas counteract host cell apoptosis. We found that the mycoplasma glycine cleavage system subunit H (GcvH) doubles as an apoptosis inhibitor. It binds to and stabilizes the ER-resident kinase Brsk2, which subsequently disturbs unfolded protein response (UPR) signaling. This disturbance leads to the inhibition of the key apoptotic molecule CHOP expression and ER-mediated intrinsic apoptotic pathway. CHOP further transduces this anti-apoptosis signal to mitochondria. Notably, GcvH N-terminal amino acid 31–35 region was necessary for its interaction with Brsk2, as well as for GcvH anti-apoptotic and potentially pro-infective activity. Our study unveils strategies by which genome-reduced bacteria exploit a limited number of genomic proteins to resist host cell apoptosis thereby enhancing pathogenesis.

## Introduction

Mycoplasmas are among the simplest prokaryotes capable of self-replication [1]. These organisms belong to the class Mollicutes, a large group of wall-less bacteria whose evolution from Gram-positive ancestors resulted in a severe genome reduction. Despite their apparent simplicity, mycoplasmas are successful pathogens in establishing persistent infections, and causing debilitating diseases in humans and a wide range of animal hosts [1,2]. Despite numerous mycoplasma diseases having a huge socio-economic significance, proper control strategies are absent, largely due to a poor understanding of their pathogenicity mechanisms [3–5].

Host apoptosis activates anti-infective immune responses and pathogen component degradation, resulting in rapid and effective pathogen clearance [6]. Apoptosis is typically triggered by extrinsic (death receptor) and/or intrinsic (mitochondrial or endoplasmic reticulum (ER)-mediated) apoptotic pathways [7]. They are carried out by Caspases, a family of cysteine-dependent aspartate-directed proteases that cleave specific target proteins [8]. Each apoptotic pathway activates its own initiator Caspase, which in turn activates the executioner Caspase-3, resulting in nuclear and cytosolic morphological changes and finally in cell death [9]. In extrinsic apoptosis, death receptor stimulation recruits and activates Caspase-8, which further processes Caspase-3 to engage apoptosis. For intrinsic apoptosis, mitochondria-mediated apoptotic signals lead to critical changes, such as a decrease in membrane potential, cytochrome c (Cyt c) release to bind apoptotic protease activating factor 1 (Apaf-1), and Caspase-9 cleavage-activation [10,11]. ER-mediated intrinsic apoptotic pathway is aroused either through Caspase-12 or through the unfolded protein response (UPR), and then the key apoptotic molecule CHOP delivers the apoptotic signals from ER to mitochondria and Caspase-9 via the B-cell lymphoma 2 (Bcl-2) family, which consists of mitochondria-associated pro-/anti-apoptotic proteins. Finally, Caspases-12 and -9 converge on Caspase-3 to initiate the death program [12–14].

Bacterial pathogens have developed various strategies to subvert host cell apoptosis to their benefit [15]. Pathogen-induced apoptosis can serve to eliminate important immune cells, or activate an inflammatory process to disrupt tissue barriers for microbial spreading in the host [16]. Microbial infections inhibit or prevent host cell apoptosis to maintain a favorable ecological niche for proliferation, and to circumvent the host antimicrobial response initiated by apoptosis [17]. The apoptosis induction or inhibition is often crucial for a successful infection within the host [18]. Understanding how pathogens modulate host cell apoptosis is indispensable for elucidating the pathogenesis of infectious diseases. Mycoplasmas have been controversial in regulating host cell apoptosis. While most mycoplasmas are known to trigger host cell apoptosis [19–21], some have evolved mechanisms to prevent it. *Mycoplasma fermentans* and *Mycoplasma penetrans* can inhibit apoptosis in murine myeloid cells, leading to malignant transformation after 4 to 5 weeks of infection, leading to rapid tumor formation when injected into nude mice [22,23]. Studies have demonstrated that *M*. *bovis* can delay apoptosis in bovine peritoneal macrophage cells (BoMac) [24]. *Mycoplasma gallisepticum* delays apoptosis in turkey tracheal organ cultures (TOC) [25]. Growing evidence suggests that mycoplasma inhibition on host cell apoptosis is not a coincidental event, but is likely to be a significant pathogenic mechanism. There are numerous cases of bacterial pathogens utilizing anti-apoptosis to cause disease [26–28]. Despite this, the anti-apoptotic role and mechanisms of mycoplasma are not well characterized. Therefore, we have focused on studying mycoplasma anti-apoptotic properties to further understand the mechanisms by which mycoplasma resists apoptosis as well as differentially regulates host apoptosis, thereby enhancing our comprehension of mycoplasma survival, replication, and dissemination strategies.

Glycine serves as a major source of single carbon units for biochemical reactions within bacterial cells, and is necessary for the biosynthesis of several important metabolites such as purines, thymidine, methionine, threonine and lipids [29,30]. The glycine cleavage system (GCS) is a multienzyme complex composed of four proteins termed P-protein (GcvP; glycine decarboxylase), H-protein (GcvH; glycine cleavage system hydrogen carrier protein, containing lipoic acid), T-protein (GcvT; aminomethyltransferase), and L-protein (GcvL; dehydrolipamide dehydrogenase), and catalyzes the oxidative cleavage of glycine, a major glycine catabolic pathway in various organisms [31]. Four GCS proteins have surfaced in many viral and bacterial pathogenesis mechanisms [32–35]. Some bacterial infections such as *Francisella tularensis*, *Brucella abortus*, or *Mycobacterium tuberculosis* are dependent on the GcvH or other GCS proteins [33,36,37]. Intriguingly, as minimalist genomic prokaryotes, ruminant and pig mycoplasmas have chosen to retain the GCS [38]. Given the critical role of GCS proteins in the pathogenicity of several bacteria, it raised the question of whether these GCS proteins also serve as mycoplasma pathogenicity factors. However, the GCS proteins have not been investigated during mycoplasmas infection.

*M*. *bovis*, a re-emerging cause of pneumonia and mastitis in cattle worldwide, has evolved strategies to delay cell apoptosis, providing a model system in this study to investigate the role and mechanism of mycoplasma pathogens hijacking host cell apoptosis [24,39,40]. Our current study uncovered for the first time that the key mycoplasmas glycine cleavage system (GCS) protein GcvH, functions as a host cell apoptosis inhibitor by targeting Brsk2 on the ER to disrupt intrinsic apoptotic pathways, thereby mediating the anti-apoptotic mechanism to promote bacterial infection. We hope that this study will lay the groundwork for a comprehensive understanding of mycoplasmas regulation on host cell apoptosis, and may offer new avenues for developing innovative approaches and novel drugs to combat mycoplasmas infection.

## Results

### 1. Anti-apoptotic effect of *M*. *bovis*

**Fig 1:**
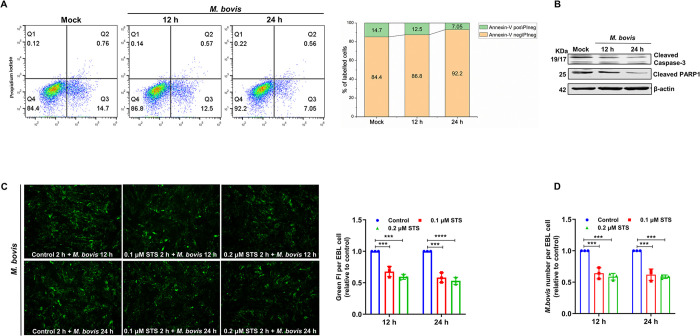
Anti-apoptotic effect of *M*. *bovis*. (A) Apoptosis analysis in *M*. *bovis*-infected cells by flow cytometry with dual Annexin V-PI cell labeling. Q4 quadrants represent intact cells (Annexin V negative\PI negative); Q3 quadrants represent early apoptotic cells (Annexin V positive\PI negative); Q2 quadrants indicate late apoptotic cells (Annexin V positive\PI positive); and Q1 quadrants indicate necrotic cells (Annexin V negative\PI positive). The graph on the right represents the percentage of apoptotic and intact cells, and the non-significant percentages of Annexin V-negative and PI-positive cells were excluded. (B) The cleaved levels of Caspase-3 and PARP1 were downregulated in *M*. *bovis*-infected EBL cells. (C-D) The percentage of cell-associated *M*. *bovis* significantly decreased in EBL cells pretreated with STS. All assays were performed with three independent experiments, and values represent the means ± SDs. Significance was assessed by one-way ANOVA with Dunnett’s multiple comparison tests relative to the control. ***, *p < 0*.*001*; ****, *p < 0*.*0001*.

We first confirmed whether the selected model system-*M*. *bovis* inhibits host cell apoptosis. Embryonic bovine lung epithelial (EBL) cells are widely used to study bovine respiratory pathogen-host interactions and bovine-specific pathogenesis, including *M*. *bovis*. We first infected EBL cells with *M*. *bovis* TJ strain at a multiplicity of infection (MOI) of 50, and used Annexin V and propidium iodide (PI) staining to analyze the percentage of live, apoptotic, and dead cells by FACS flow cytometry. Compared to mock-infected cells, *M*. *bovis* infection decreased the EBL cell apoptosis level (early apoptosis Q3 + late apoptosis Q2), especially the early apoptotic cells percentage dropped from 14.7% in the control group to 7.05% at 24 hpi (Fig 1A). Terminal deoxynucleotidyl transferase-mediated dUTP-biotin nick end labeling (TUNEL) assay could identify apoptotic cells that undergo DNA breakage by adding FITC-labeled dUTP to the fragmented DNA ends. Cells even at the early stages of apoptosis could be visualized by TUNEL detection. Consistent with flow cytometry findings, TUNEL signals (green fluorescence) revealed less DNA damage and death in *M*. *bovis*-infected cells than in mock-infected cells (S1A Fig). Apoptosis is a highly regulated process involving a series of cysteine protease caspase cascade activation reaction events, of which Caspase-3 is the key terminal caspase and its cleavage-activation to cleave the substrate Poly (ADP-Ribose) Polymerase 1 (PARP1) is an important apoptotic marker [41,42]. Their cleavage decreased with *M*. *bovis* infection (Fig 1B). These data suggest that *M*. *bovis* infection inhibits host EBL cell apoptosis.

Furthermore, staurosporine (STS, an apoptotic inducer) activates Caspase-3 cleavage and has been proven effective in inducing apoptosis of cultured mammalian cells [43]. We incubated EBL cells with or without *M*. *bovis* at an MOI of 50 for 18 h, and then with 0.25 μM STS for a further 6 h. It was discovered that pre-infection with *M*. *bovis* followed by incubation with STS induced a significant reduction in apoptosis compared to that induced by incubation with STS alone, indicating that *M*. *bovis* also actively diminished the STS-induced EBL cell apoptosis (S1B Fig).

To explore the link between apoptosis and *M*. *bovis* infection of EBL cells, we treated EBL cells with 0.1 or 0.2 μM STS for 2 h to induce cell apoptosis (S1C Fig), and then removed STS to allow infection with *M*. *bovis* at an MOI of 50. Next, we employed an indirect immunofluorescence assay (IFA) targeting *M*. *bovis* to assess whether STS-induced apoptosis impacted the number of mycoplasmas per EBL cells. We visualized cell nuclei by DAPI (4’,6-diamidino-2-phenylin-dole) to quantify cell amount [44], and measured the green fluorescence intensity (FI, *M*. *bovis* signal) in the corresponding view field (Figs S1D and 1C). The reduction in the relative levels of green FI per cell (green FI/cell number) compared to that of the untreated cells infected with *M*. *bovis*, suggests that the percentage of cell-associated *M*. *bovis* decreased significantly upon inducing apoptosis (Fig 1C). These results were further confirmed by TaqMan detection of *M*. *bovis* genomic DNA (Fig 1D).

Taken together, these results indicate that the anti-apoptotic effect of *M*. *bovis* promotes EBL cell infection.

### 2. *M*. *bovis* glycine cleavage system H protein (GcvH) is an anti-apoptotic protein

**Fig 2:**
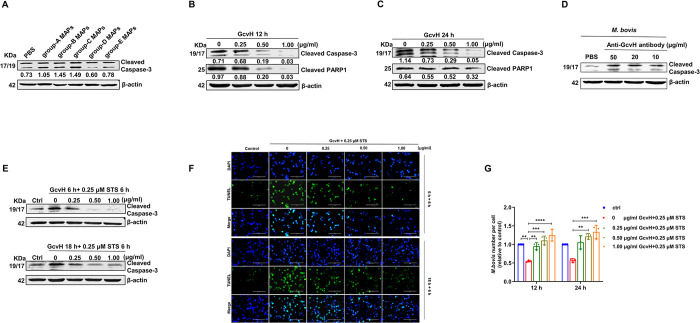
*M*. *bovis* encodes an anti-apoptotic protein GcvH. (A) The group-D MAPs decreased cleaved Caspase-3 levels by Western blotting analysis. (B-C) Western blotting revealed that the cleaved levels of Caspase-3 and PARP1 were reduced following GcvH protein incubation in EBL cells. (D) GcvH blockage increased *M*. *boivs* apoptotic effect on EBL cells. *M*. *bovis* was incubated with the purified antibodies against GcvH (10, 20 and 50 μg/ml) or PBS for 30 min and then added to EBL cells. The cleaved Caspase-3 level in EBL cells was determined by Western blotting. (E-F) GcvH lowered or prevented STS-induced apoptosis in EBL cells. EBL cells were treated with 0, 0.25, 0.5 or 1.00 μg/ml GcvH for 6 or 18 h, and then with 0.25 μM STS. Cells were collected for Western blotting analysis of cleaved Caspase-3 level (E), or for TUNEL detection (F). (G) EBL cells were treated as described in panel E and then incubated with *M*. *bovis* for 12 h. The number of *M*. *bovis* per EBL cells was surveyed by TaqMan qPCR. The protein levels were quantified by ImageJ and normalized to β-actin (A, B and C). The data are presented as the means ± SDs from three independent experiments, and significance was assessed by one-way ANOVA with Tukey’s multiple comparison test. **, *p < 0*.*01*; ***, *p < 0*.*001*; ****, *p < 0*.*0001*.

We suspected that *M*. *bovis* membrane-associated proteins (MAPs) have a role in preventing host cell apoptosis. Interactions of the MAPs with the host cells are thought to be one of the major factors in mycoplasma pathogenesis [45]. Thus, we attempted to screen MAPs for proteins with anti-apoptotic properties. *M*. *bovis* MAPs were extracted, and then separated into five groups (designated as group-A, -B, -C, -D and -E) using AKTA molecular sieve (S2A and S2B Fig). Due to the large number of mycoplasma MAPs, molecular sieve grouping can help to narrow the screening range. We found that group-D MAPs (1 μg/ml) decreased the cleaved Caspase-3 level in 12 h without affecting EBL cell viability (Figs 2A and S2C). The data indicated that group-D MAPs had an anti-apoptotic effect, and thus a mass spectrometry (MS) analysis was performed on this group. Forty-eight *M*. *bovis* membrane proteins were identified, and several candidates were chosen to investigate apoptotic-regulation based on their matching scores and gene annotations. Among these candidates, MS data showed matches to 4 peptides of glycine cleavage system key enzyme H (GcvH) with approximately 35% sequence coverage (S2D and S2E Fig). GcvH was the only one that inhibited host cell apoptosis, and was thus selected for subsequent studies (S2F Fig).

GcvH is a component of the conserved glycine cleavage system (GCS) that converts glycine into one-carbon units [46]. By purifying the GcvH fusion protein with an MBP tag and removing the MBP tag by protease cleavage, we obtained the GcvH protein (S2G and S2H Fig). Different concentrations (0.25, 0.5 or 1 μg/ml) of GcvH protein were subsequently found to inhibit Caspase-3 and PARP1 cleavage in EBL cells in a dose-dependent manner in 12 and 24 h, compared to the 0 μg/ml GcvH control group (Fig 2B and 2C). The selected concentrations of GcvH had no significant cytotoxicity on EBL cell viability (S2I Fig). These data indicate that *M*. *bovis* GcvH inhibits EBL cell apoptosis. Finally, we purified a specific antibody against GcvH from murine serum anti-GcvH to preincubate *M*. *bovis* for blocking its surface GcvH, and observed that these *M*. *bovis* increased EBL cell apoptosis compared to untreated *M*. *bovis* infection, confirming that GcvH on *M*. *boivs* cells surface also inhibits host cell apoptosis (Fig 2D).

Overall, these findings suggest that we have successfully identified an *M*. *bovis* protein opposing EBL cell apoptosis.

### 3. GcvH blocks Caspase-3 activator-induced apoptosis and promotes *M*. *bovis* infection of EBL cells

To further characterize the anti-apoptotic effect of GcvH, EBL cells were pretreated with different concentrations (0, 0.25, 0.5 or 1 μg/ml) of GcvH protein for 6 or 18 h, followed by incubation with 0.25 μM STS for a further 6 h. Western blot analysis of Caspase-3 cleavage activation showed that GcvH pretreatment diminished or blocked the STS-induced apoptosis in EBL cells (Fig 2E). The CCK-8 assay revealed that GcvH mitigated the detrimental effect of STS on EBL cell viability (S2J Fig). TUNEL detection also showed that GcvH reduced or prevented STS-induced apoptosis by comparing the relative change in green fluorescence intensity (Fig 2F). These findings imply that GcvH actively impeded Caspase-3 activation to prevent apoptosis in STS-treated EBL cells. Incubation of EBL cells with STS (0 μg/ml GcvH + 0.25 μM STS) followed by *M*. *bovis* infection (MOI = 50) showed that STS-induced apoptosis reduced the percentage of mycoplasmas per EBL cells, compared to untreated cells (Fig 2I). However, pretreated with GcvH followed by exposure to STS (0.25/0.5/1.0 μg/ml GcvH + 0.25 μM STS) increased the number of mycoplasmas per EBL cells compared to cells exposed to STS alone (Fig 2G), suggesting that GcvH actively hinders the apoptosis initiation in STS-treated EBL cells, and promotes the infection of EBL cells with *M*. *bovis*.

### 4. GcvH resists host cell apoptosis through the intrinsic apoptotic signaling

**Fig 3:**
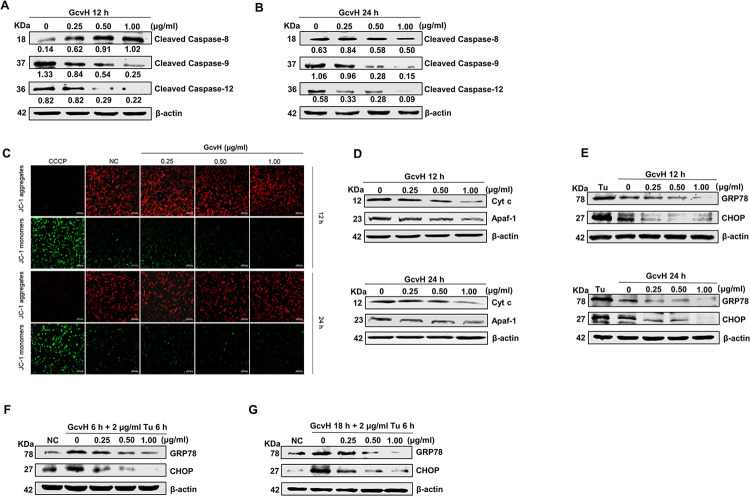
GcvH resists host cell apoptosis through intrinsic apoptotic signaling. (A-B) EBL cells were mocked or incubated with GcvH for 12 or 24 h. Cell lysates were analyzed by western blotting for cleaved Caspase-8, -9, -12 and β-actin. The protein levels were quantified by ImageJ and normalized to β-actin. (C) The mitochondrial membrane potential (MMP) of GcvH-incubated EBL cells was measured by the JC-1 probe, CCCP as positive control. (D) The levels of Cyt c and Apaf-1 in GcvH-incubated EBL cells were evaluated by Western blotting. (E) GcvH treatment decreased the levels of GRP78 and CHOP. (F-G) Tunicamycin (Tu)-induced increases in GRP78 and CHOP expression were reduced or even abolished by GcvH.

Apoptosis is typically triggered by extrinsic (death receptor-mediated) and/or intrinsic (mitochondrial or endoplasmic reticulum (ER)-mediated) apoptotic pathways, which are initiated separately by Caspase-8, -9 and -12 and converge at Caspase-3 [12,47]. To elucidate the pathway by which GcvH prevents host cell apoptosis, western blot assays were performed. The results revealed an increase in Caspase-8 cleavage activation at 12 h and a slight change at 24 h, along with a significant reduction in both Caspase-9 and -12 cleavage activation at both time points, indicating that GcvH counteracts EBL cell apoptosis by suppressing the intrinsic apoptotic pathway (Fig 3A and 3B).

To further verify the inhibition of GcvH on the mitochondria-mediated intrinsic apoptotic pathway, we employed the JC-1 (5, 5’, 6, 6’-tetrachloro-1, 1’, 3, 3’-tetraethylbenzimidazolylcarbocyanine iodide) probe to measure the mitochondrial membrane potential (MMP). The MMP loss is often associated with the commitment to mitochondria-mediated apoptosis [48]. Healthy mitochondria with high membrane potential assemble JC-1 and emit red fluorescence, while apoptotic mitochondria with a low membrane potential cause it to appear as a monomer and emit green fluorescence. Carbonyl cyanide m-chlorophenyl hydrazine (CCCP) is a classical positive control that increases the proton permeability of the mitochondrial membrane to disrupt the MMP. As shown in Fig 3C, the reduced green fluorescence showed an elevated MMP in EBL cells after 12 and 24 h of GcvH stimulation compared to the cell control group, confirming that GcvH hindered the mitochondria-mediated apoptotic pathway in EBL cells. Further evidence that GcvH inhibited the mitochondria-mediated intrinsic apoptotic pathway in EBL cells was found in the decreased protein levels of cytochrome c (Cyt c) and apoptotic protease activating factor 1 (Apaf-1), key pro-apoptotic molecules in this apoptotic pathway (Fig 3D).

The unfolded protein response (UPR) was discovered to trigger ER-mediated apoptosis in addition to Caspase-12 [12]. We next detected how GcvH tuned the host UPR to determine whether it suppressed the ER-mediated intrinsic apoptotic pathway. GcvH was observed to decrease the protein levels of glucose-regulated protein 78 (GRP78) and C/EBP homologous protein (CHOP), which are core molecules of UPR activation and UPR-mediated apoptotic pathway respectively (Fig 3E). In contrast, tunicamycin (Tu), a routinely used UPR inducer, caused the opposite changes at both GRP78 and CHOP protein levels (Fig 3E). Notably, GcvH attenuated and even reversed the elevation of GRP78 and CHOP protein expression induced by Tu (Fig 3F and 3G). Consistently, CCK-8 assays showed that GcvH alleviated the detrimental impact of Tu on EBL cell viability (S2K Fig). These results exhibit that GcvH inhibits ER-associated (mediated by Caspase-12 or UPR) intrinsic apoptotic pathway.

Collectively, GcvH counteracts EBL cell apoptosis by interfering with intrinsic apoptotic pathways.

### 5. GcvH disturbs three UPR branches to inhibit ER-mediated intrinsic apoptosis

**Fig 4:**
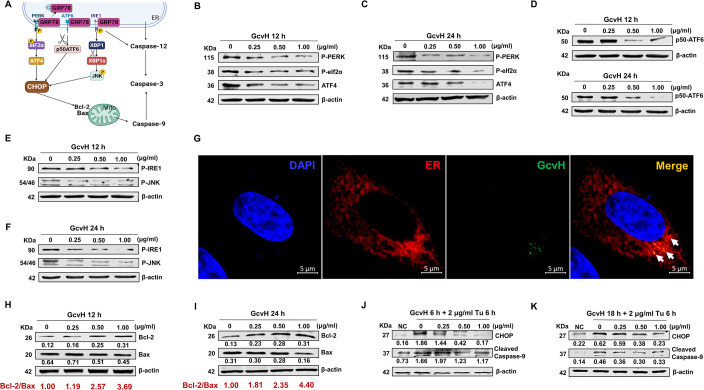
GcvH disturbs three UPR branches to block ER-mediated apoptosis. (A) Schematic showing CHOP activated by the three UPR branches in the ER. (B-C) The inhibition of the PERK branch in EBL cells following GcvH incubation was confirmed by Western blotting of the levels of phosphorylation-PERK, -eIF2α and ATF4. (D) The downregulation of ATF6 branch in EBL cells with GcvH addition was determined by Western blotting of ATF6 cleavage (p50 ATF6). (E-F) The suppression of the IRE1 pathway in EBL cells following GcvH incubation was obtained by Western blotting of the phosphorylation level of IRE1 and JNK. (G) EBL cells were incubated with 1 μg/ml GcvH protein for 12 h, and then intracellular GcvH protein (green) was localized using the purified specific anti-GcvH antibody conjugated with an anti-mouse fluorescent secondary antibody. The EBL cell’s endoplasmic reticulum (ER, red) was labeled by transfecting with the plasmid pDsRed2-ER. DAPI was used to stain cellular nuclei (blue). (H-I) GcvH-induced enhancement of the Bcl-2/Bax ratio (red characters beneath the WB bands) was verified by Western blotting for the level of Bcl-2 and Bax. (J-K) EBL cells were incubated with GcvH (0, 0.25, 0.5 and 1 μg/ml) for 6 or 18 h, and followed by an addition of 2 μg/ml Tu for an additional 6 h. Cell lysates were tested by western blotting for CHOP, cleaved Caspase-9 and β-actin. The protein levels were quantified by ImageJ and normalized to β-actin (H, I, J and K).

As illustrated in Fig 4A, the UPR consists of three signaling pathways: (i) the protein kinase R-like endoplasmic reticulum kinase (PERK), (ii) the activating transcription factor 6 (ATF6), and (iii) the inositol-requiring enzyme 1 (IRE1). These three UPR branches lead to CHOP (the core molecule of the UPR-mediated apoptotic pathway) transcription to trigger ER-associated apoptosis. Activated PERK phosphorylates eukaryotic translation initiation factor 2 alpha (eIF2α), which preferentially translates the activating transcription factor 4 (ATF4), thereby promoting CHOP expression. ATF6 is proteolytically activated to its active 50-kDa form (p50-ATF6) in the Golgi apparatus to upregulate CHOP. Furthermore, IRE1 activation converts unspliced X-box binding protein 1 (XBP1) mRNA into active XBP1s, which activates the c-Jun N-terminal kinase (JNK) phosphorylation to enhance CHOP production in the nucleus [49].

To determine which signaling pathway GcvH employed to suppress the UPR for blocking the ER-mediated apoptotic pathway, we measured PERK and eIF2α phosphorylation in EBL cells after exposure to GcvH protein. GcvH reduced both PERK and eIF2α phosphorylation (Fig 4B and 4C), as well as the transcription and translation of their downstream component ATF4 (Figs 4B, 4C and S3A). These data indicate that GcvH inhibits PERK signaling. We subsequently discovered a decrease in the ATF6 cleavage-activated form p50-ATF6, indicating that GcvH also restrains the ATF6 signaling pathway (Fig 4D). Finally, we observed that GcvH lowered the phosphorylation of both IRE1 and JNK (Fig 4E and 4F). Similarly, XBP1, a key molecule downstream of IRE1, was also diminished in its active form XBP1s (S3B Fig). These results imply that GcvH also suppresses the IRE1 signaling pathway.

Altogether, these findings suggest that GcvH suppresses all three UPR signaling pathways to prevent the UPR activation, and thereby inhibiting ER-mediated apoptosis.

### 6. GcvH transmits anti-apoptotic signaling from the endoplasmic reticulum to mitochondria

To check the intracellular localization of GcvH, we employed the purified mouse-specific antibody against GcvH conjugated with a fluorescent secondary antibody to visualize GcvH in EBL cells after incubation with GcvH protein or *M*. *bovis* (MOI = 50). The EBL cell ER was labeled by transfecting with the plasmid pDsRed2-ER. We observed that both prokaryotically expressed GcvH and *M*. *bovis* membrane component GcvH are located to the ER, indicating that GcvH targets ER to inhibit ER-associated apoptosis (Figs 4G and S3C).

The B-cell lymphoma 2 (Bcl-2) family proteins located on the mitochondrial membrane, which consists of pro- and anti-apoptotic proteins, play a key role in regulating mitochondria-mediated apoptosis [50]. Previous studies have demonstrated that reduced CHOP expression shifts Bcl-2 family towards anti-apoptosis, which in turn represses mitochondria-mediated intrinsic apoptosis [51,52] (Fig 4A). We next tested this possibility in the GcvH anti-apoptotic mechanism, given that GcvH targets ER and decreases CHOP levels. Therefore, we investigated whether the Bcl-2 family was shifted to anti-apoptosis upon GcvH incubation. Bcl-2 and Bcl-2 associated X protein (Bax) are key anti- and pro-apoptotic members of the Bcl-2 family respectively. The ratio of Bcl-2/Bax is a significant indicator for evaluating the anti-/pro-apoptotic direction of the Bcl-2 family [53]. We detected Bcl-2 and Bax protein levels in EBL cells after GcvH incubation and calculated their ratio. GcvH (0, 0.25, 0.5 and 1.0 μg/ml) dose-dependently increased the Bcl-2/Bax ratio from 1.0 to 3.69 at 12 h and from 1.0 to 4.4 at 24 h, indicating that GcvH shifted the Bcl-2 family to be anti-apoptosis. These data imply that the anti-apoptotic signaling of GcvH may be transmitted by CHOP from the ER to the Bcl-2 family and mitochondria (Fig 4H and 4I). Subsequently, we upregulated CHOP expression with the UPR activator Tu to block the transfer of GcvH anti-apoptotic signal from ER to the mitochondria. As shown in Fig 4J and 4K, 1 μg/ml GcvH pretreatment of EBL cells for 6 or 18 h followed by Tu incubation for 6 h, the CHOP level was comparable to that of the untreated cell, suggesting that Tu blocked CHOP transmitting GcvH anti-apoptotic signal from ER to mitochondria. We interestingly found that GcvH no longer inhibited caspase-9 cleavage activation, indicating that the signal for GcvH inhibition on the mitochondrial apoptotic pathway originates from ER.

Summarily, these results suggest that GcvH targets ER to disturb the ER-associated apoptotic pathway and then signals mitochondria, ultimately resisting host cell apoptosis through these two intrinsic apoptotic pathways.

### 7. Brsk2 is essential for the anti-apoptotic effect of GcvH

**Fig 5:**
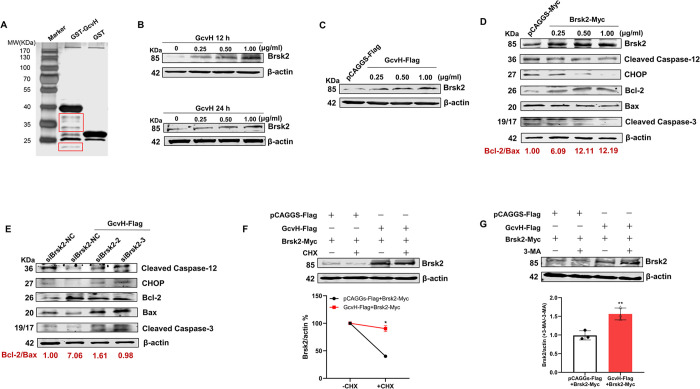
BRSK2 is essential for the anti-apoptotic effect of GcvH. (A) Screening for the host proteins interacting with GcvH by the GST pull-down assay. (B-C) GcvH-induced promotion of Brsk2 expression was confirmed by Western blotting of the Brsk2 and β-actin levels in EBL cells with GcvH protein incubation (B) or with transfection of the GcvH-Flag plasmid (C). (D) The inhibition of ER-associated intrinsic apoptotic pathway and apoptosis in EBL cells transfected with the GcvH-Flag plasmid were ascertained by Western blotting of the levels of cleaved Caspase-12, -3, CHOP, Bcl-2, Bax, and β-actin. (E) Knockdown of Brsk2 disrupted the inhibitory effect of GcvH on EBL cell apoptosis. (F) GcvH stabilized Brsk2 by preventing its degradation. EBL cells coexpressing Brsk2-Myc and GcvH-Flag or empty vector control (Flag) were treated with the protein synthesis inhibitor cycloheximide (CHX; 75 μg/ml) for 12 h. Brsk2 and β-actin levels were determined by Western blotting analysis. Densitometry analysis of Brsk2 levels relative to actin (Brsk2/actin) normalized to no CHX treatment. (G) GcvH augmented Brsk2 stability by diminishing the autophagic degradation of Brsk2. EBL cells coexpressing Brsk2-Myc and GcvH-Flag or empty vector control (Flag) were treated with an autophagy inhibitor 3-MA (5 mM, 12 h). Brsk2 and β-actin levels were determined by Western blotting analysis. Densitometry analysis of Brsk2 levels relative to actin (Brsk2/actin) in 3-MA-treated or -untreated EBL cells. The ratio of 3-MA-treated (Brsk2/actin) was normalized to 3-MA-untreated (Brsk2/actin). The data represented the means ± SDs of the results from three independent experiments. Significance was assessed by a two-tailed Student’s *t* test. *, *p < 0*.*05*; **, *p < 0*.*01*.

To gain a deeper understanding of how GcvH inhibits host cell apoptosis, we identified the key host molecules involved. After generating and purifying the GST-tagged GcvH fusion protein (S4A Fig), we performed a GST pull-down assay (Fig 5A). Four distinct protein bands were pulled down and analyzed by MS, which identified four host proteins including BR serine/threonine-protein kinase 2 (Brsk2). Given the presence of Brsk2 in the ER, we hypothesized that GcvH impaired the ER-mediated intrinsic apoptotic pathway through Brsk2. To test this point, we incubated EBL cells with GcvH (0, 0.25, 0.5 and 1.0 μg/ml) and discovered an enhanced Brsk2 expression (Fig 5B), which was further confirmed by assessing EBL cells transfected with GcvH-flagged plasmids (Fig 5C). Subsequently, we observed that increased Brsk2 expression in EBL cells transfected with Brsk2-Myc plasmids inhibited the ER-associated intrinsic apoptotic pathway and apoptosis, as evidenced by reduced Caspase-12 and -3 cleavage activation, decreased CHOP expression, while increased Bcl-2/Bax ratio (Fig 5D). These findings indicated that GcvH interacted with Brsk2 and promoted its expression, which in turn inhibited host cell apoptosis. Moreover, we constructed three specific siRNAs targeting bovine Brsk2, of which siBrsk2-2 and -3 significantly reduced the Brsk2 expression in EBL cells (S4B Fig). In GcvH-overexpressed EBL cells, the Brsk2 knockdown using these two siRNAs disrupted the inhibition of GcvH on Caspase-12 and -3 cleavage, boosted CHOP expression, and significantly diminished the Bcl-2/Bax ratio, compared to that in control siRNA siBrsk2-NC knockdown (Fig 5E). According to these findings, Brsk2 knockdown nearly eliminates GcvH anti-apoptotic action, suggesting its crucial role in GcvH anti-apoptotic function.

### 8. GcvH stabilizes the Brsk2 protein

To understand the mechanism by which GcvH increases Brsk2 protein levels, we first tested whether GcvH regulates Brsk2 mRNA transcription. As shown in S4C Fig, there was no increase in Brsk2 mRNA levels in EBL cells transfected with GcvH-Flag. We hence hypothesized whether GcvH affected Brsk2 protein stability based on the presence of a GcvH-Brsk2 interaction. We then monitored Brsk2 protein decay in EBL cells transfected or not transfected with GcvH-Flag plasmid, upon the addition of the protein synthesis inhibitor cycloheximide (CHX). Brsk2 protein was detected at higher levels in EBL cells transfected with GcvH-Flag, even up to 12 h of protein synthesis shutdown, than in the vector control (Fig 5F). Brsk2 levels decreased by 10% of CHX treatment in the presence of GcvH, whereas a reduction of 60% occurred in the absence of GcvH (Fig 5F). These results show that the expression of GcvH protein stabilizes the Brsk2 protein by preventing its degradation.

The ubiquitin-proteasome system and autophagy are two major intracellular pathways for protein degradation [54]. We next investigated which process is crucial in GcvH-mediated Brsk2 upregulation using the proteasomal inhibitor MG-132 and the autophagy inhibitor 3-MA, respectively. Brsk2 levels increased in GcvH-expressing cells treated with the autophagy inhibitor (Fig 5G), whereas a decrease in the proteasomal inhibitor-treated cells (S4D Fig). These results indicate that GcvH diminishes Brsk2 autophagic degradation, increasing its stability and levels.

### 9. GcvH N-terminal mutations (aa 31 to 35) abolish its interaction with Brsk2

**Fig 6:**
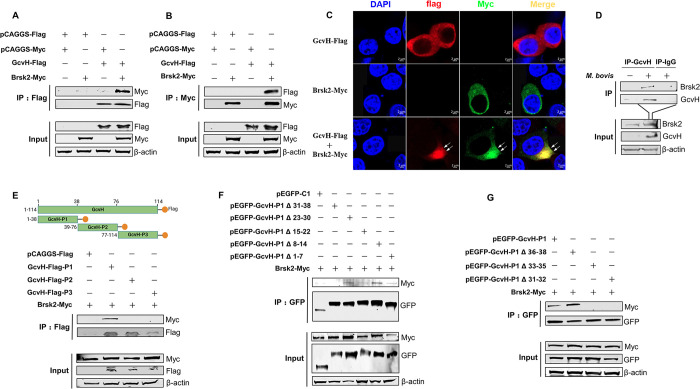
GcvH N-terminal mutations (aa 31 to 35) abolish its interaction with Brsk2. (A-B) GcvH-Flag coimmunoprecipitated with Brsk2-Myc. Hela cells were transfected with GcvH-Flag or Brsk2-Myc, or the control plasmids pCAGGS-Flag or pCAGGS-Myc for 36 h. Cell lysates were collected for Co-IP with beads conjugated with anti-Flag antibody (A) and Myc antibody (B). (C) Colocalization of the GcvH and Brsk2 proteins. (D) Hela cells were infected with *M*. *bovis* and the interaction of endogenous Brsk2 and GcvH protein in the cell lysates was detected by coimmunoprecipitation with antibody against GcvH proteins rather than IgG and protein A-conjugated beads. (E) The fragment of GcvH aa 1–38 interacted with the Brsk2 protein. Upper: Schematic of the GcvH truncates tagged with Flag and locations of the aa residues are noted. Lower: Hela cells were co-transfected with Brsk2-Myc and truncated GcvH constructs, and the cell lysates were collected for Co-IP assays using beads conjugated with anti-Flag antibody for Flag-tagged GcvH truncates or with Myc antibody for Myc-tagged Brsk2. (F) The GcvH plasmid lacking the aa 31 to 38 did not immunoprecipitate with the Brsk2. Hela cells were cotransfected with Brsk2-Myc and the five GcvH-P1 deletion constructs. Cell lysates were collected for Co-IP assays using beads conjugated with anti-GFP antibodies. (G) The GcvH plasmid lacking the aa 31 to 32 or 33 to 35 did not immunoprecipitate with the Brsk2. Hela cells were cotransfected with Brsk2-Myc and the three GcvH-P1 deletion constructs, and then collected to perform a Co-IP assay.

To further validate the specific interaction of GcvH with Brsk2, Co-immunoprecipitation (Co-IP) was performed on Hela cells transfected with plasmids expressing Flagged GcvH and Myc-tagged Brsk2 together or alone. Reciprocal interactions were observed in the Co-IP assays using anti-Flag or anti-Myc antibodies (Fig 6A and 6B). Confocal microscopy was also employed and showed the colocalization of GcvH with Brsk2 in the cytoplasm of Hela cells (Fig 6C). These results indicate that *M*. *bovis* GcvH protein interacts with Brsk2 in cells.

Also, we asked whether endogenous Brsk2 interacts with GcvH protein during *M*. *bovis* infection. EBL cells were infected or uninfected with *M*. *bovis* at an MOI of 50 for 24 h, we performed the Co-IP assay with the cells lysates and beads conjugated with IgG or antibodies to GcvH protein. Endogenous Brsk2 was immunoprecipitated with GcvH in *M*. *bovis*-infected cell lysates (Fig 6D). These results determine that endogenous Brsk2 interacts with *M*. *bovis* GcvH protein during *M*. *bovis* infection.

As illustrated in Fig 6E, truncated GcvH constructs with a Flag tag at their C-terminus were generated to identify the key domain in GcvH responsible for its interaction with Brsk2. Only the GcvH-P1 construct (N-terminal amino acids (aa) 1 to 38) was immunoprecipitated with Brsk2, suggesting it is responsible for this interaction (Fig 6E). A series of deleted GcvH-P1 (Δ1–7, Δ8–14, Δ15–22, Δ23–30 and Δ31–38 aa) were then constructed for Co-IP with Brsk2, the immunoprecipitation of GcvH with brsk2 was not observed after deleting the aa 31 to 38 (Fig 6F). Within this critical region aa 31 to 38, the elimination of amino acids 31–32 or 33–35 prevented GcvH-P1 from precipitating Brsk2 (Fig 6G). These findings implicate that amino acids at positions 31 to 35 in the GcvH N-terminal region are critical for GcvH interaction with host Brsk2.

### 10. The interaction with Brsk2 is required for the anti-host apoptotic action of GcvH

**Fig 7:**
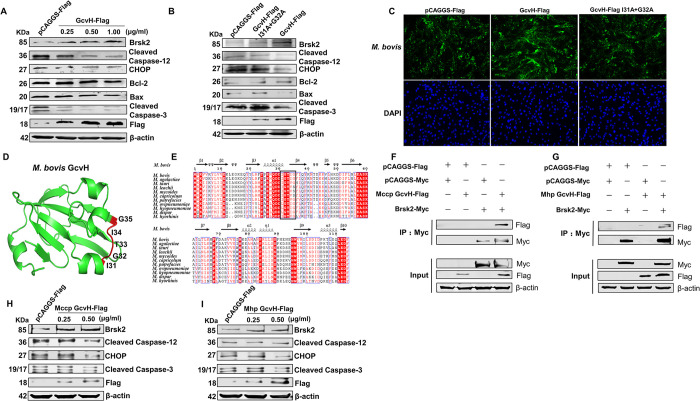
The interaction with Brsk2 is required for the anti-host apoptotic action of GcvH. (A) Transfection of the GcvH-Flag plasmid in EBL cells enhanced Brsk2 expression while hindering cell apoptosis. (B) GcvH had no anti-apoptotic effects on host cells when it lost its binding to Brsk2. (C) GcvH did not promote *M*. *bovis* infection of EBL cells when it lost the interaction with Brsk2. EBL cells were treated as stated in panel B and then subjected to detect cell-associated *M*. *bovis* by an IFA. (D) The PDB structure of *M*. *bovis* GcvH was predicted by the Alphafold network. Full-length GcvH protein (green) and its key amino acid sites 31–35 interacting with Brsk2 are shown here in red. This structure was visualized in Pymol software. (E) A multiple sequence comparison of GcvH between GCS-containing mycoplasmas. (F-G) Both Mccp GcvH-Flag and Mhp GcvH-Flag coimmunoprecipitated with Brsk2-Myc. (H-I) Both Mccp GcvH-Flag and Mhp GcvH-Flag transfection decreased host cell apoptosis.

We further analyzed whether GcvH resists host cell apoptosis by interacting with Brsk2. A transfection of GcvH-Flag plasmid was determined to promote Brsk2 expression, inhibit CHOP expression, and reduce Caspase-12 and -3 activation, thereby inhibiting host cell apoptosis (Fig 7A). Subsequently, we transfected EBL cells with a mutant plasmid of GcvH-Flag in which both key amino acid sites 31 and 32 interacting with Brsk2 were mutated to alanine (A). It was found that mutated GcvH neither increased the Brsk2 expression, nor inhibited the CHOP expression, nor decreased Caspase-12 and -3 activation compared to the control cells transfected with pCAGGS-Flag, indicating that GcvH no longer had an anti-apoptotic effect on host cells when it loses its interaction with Brsk2 (Fig 7B). In addition, we conducted IFA and TaqMan qPCR assays and found that GcvH-Flag transfection enhanced infection of EBL cells with *M*. *bovis*, compared to the control plasmid pCAGGS-Flag transfection. However, the detection of *M*. *bovis* in EBL cells transfected with the mutant GcvH-Flag plasmid was comparable to that of EBL cells transfected with the control plasmid (Figs 7C and S4E). These results suggest that the interaction of GcvH with Brsk2 is necessary for its anti-apoptotic effect and promotes the infection of EBL cells with *M*. *bovis*.

### 11. Mycoplasma GcvH interacts with host Brsk2 and inhibits host cell apoptosis

The structure of the *M*. *bovis* GcvH protein was predicted by the Alphafold network as illustrated in Fig 7D, and its key amino acid sites 31–35 interacting with Brsk2 are shown in red [55]. We performed a multiple sequence comparison of GcvH between several common mycoplasmas that infect ruminants and pigs, given that GCS is currently only found in ruminant and pig mycoplasmas. We discovered that this critical amino acid region is highly conserved, as shown in the square of Fig 7E. It was hypothesized that the interaction between mycoplasma GcvH and host Brsk2 is conserved. To this end, we selected *Mycoplasma capricolum subspecies capripneumoniae* (Mccp) and *Mycoplasma hyopneumoniae* (Mhp), which infect ruminants and pigs respectively, as representatives to validate the GcvH-Brsk2 interaction. The Co-IP assay results revealed that both Mccp and Mhp GcvH interacted with host Brsk2 in Hela cells (Fig 7F and 7G), implying the interaction of mycoplasma GcvH with host Brsk2 may be conserved.

Since the interaction with Brsk2 is required for *M*. *bovis* GcvH anti-apoptotic action, we subsequently checked whether Mccp and Mhp GcvH had anti-apoptotic effects on host cells. We constructed plasmids tagged with a flag for GcvH from Mccp and Mhp, which were subsequently transfected into goat tracheal epithelial (GTE) cells and porcine tracheal epithelial (PTE) cells. As shown in Fig 7H and 7I, both GcvH of Mccp and Mhp up-regulated Brsk2 protein levels, reduced CHOP, cleaved Caspase-12 and -3 levels in host cells, exhibiting anti-apoptotic action. Therefore, we concluded that the novel function of GcvH described in this study is not unique for *M*. *bovis*, and is likely to be a conserved function of mycoplasmas GcvH.

## Discussion

**Fig 8:**
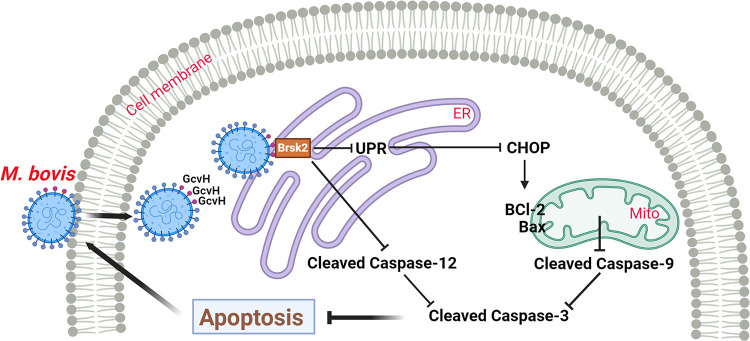
Model depicting the promotion of *M*. *bovis* epithelial infection by GcvH anti-host cell apoptosis. *M*. *bovis* attaches to cells, followed by its membrane protein GcvH binding to the host Brsk2 to resist the ER-mediated apoptotic signaling, which is transmitted to mitochondria via CHOP. GcvH promotes *M*. *bovis* infection by preventing host cell apoptosis through these two intrinsic apoptotic pathways.

Pathogenicity is the outcome of multiple biological processes that enable pathogens to survive, multiply and persist in an immunocompetent host. This is especially true for mycoplasmas, which usually cause persistent infections. Anti-host apoptosis has been evidenced as an important pathogenic mechanism in the establishment and persistence of infection by pathogens. Although documented in several species [22,25,40], the anti-apoptotic properties of mycoplasmas and their role in pathogenesis are still poorly understood. In this study, we utilized *M*. *bovis* as a model and uncovered for the first time that the key mycoplasma glycine cleavage system (GCS) protein GcvH, functions as a host cell apoptosis inhibitor by targeting Brsk2 on the ER to disrupt intrinsic apoptotic pathways, thereby mediating the anti-apoptotic mechanism to promote bacterial infection (Fig 8).

Anti-apoptotic regulation is a common bacterial survival, proliferation and infection strategy [17,56]. Previous studies on *Helicobacter pylori* (Hp) showed that Hp inhibits gastric epithelial cell apoptosis to enhance its stomach colonization [28]. *Edwardsiella tarda* prevents cell apoptosis in teleost for intracellular survival [57]. Our study indicates that host cell apoptosis can reduce the number of cell-associated mycoplasmas, suggesting a detrimental effect on *M*. *bovis* infection. Whether the anti-apoptotic properties of GcvH may contribute to *M*. *bovis* pathogenesis remains to be further investigated.

There is currently no consensus on the correlation between *M*. *bovis* infection and host apoptosis. While some studies suggested that *M*. *bovis* infection triggers host cell apoptosis, others indicated that *M*. *bovis* does not modulate or inhibit apoptosis [40,58–60]. We surmise that this discrepancy may be strain-dependent, as demonstrated by Maina *et al*. that the bovine isolate strain Mb1 inhibits host macrophage apoptosis while the bison isolate strain Mb304 does not [24]. The cell type being infected may play a role, as observed by Josi *et al*. that *M*. *bovis* L22/93 did not impact or even suppress apoptosis in bovine embryonic turbinate (PECT) cells and bovine mammary epithelial cells (bMec), but induced apoptosis in Madin-Darby bovine kidney (MDBK) cells [61]. Additionally, the multiplicity of infection (MOI) could influence *M*. *bovis* regulation on apoptosis, as indicated by Liu *et al*. that infection of bMEC at a high MOI of 1000 induced apoptosis, whereas it did not at low MOIs of 1:1.3 or 1:9 [60]. The diverse responses observed in different studies highlight the complexity of *M*. *bovis*-host cell interactions, and underscore an arms race between *M*. *bovis* and host cells.

GCS is a loosely associated four-subunit enzyme complex (GcvH, GcvP, GcvT and GcvL) that catalyzes the reversible oxidation of glycine to form 5, and 10-methylenetetrahydrofolate, which serves as a one-carbon donor utilized in the production of serine, thymidine, and purines [62]. Therefore, this pathway is expected to contribute to pathogen fitness in host compartments where these metabolites, such as serine, are limiting [32]. Notably, the GCS is also predicted to be a virulence factor associated with virulence and persistent infection in a wide range of bacteria [63]. It was required for the pathogenesis of virulent *Francisella tularensis* in a murine model, and deletion of its GcvT subunit delayed mortality and lowered bacterial burden [32]. After mutation in the GCS GcvP of the protozoan parasite *Leishmania* caused unusually low pathogenicity in experimental animals [64]. In the present study, we identified the GCS subunit GcvH as a potential virulence factor in *M*. *bovis*, probably due to its anti-apoptotic activity that promotes *M*. *bovis* infection of host cells. The recent development of new genetic tools to manipulate the *M*. *bovis* genome may help to further investigate the anti-apoptotic properties of GcvH in vivo [65].

A previous study showed that *M bovis* conserved protein P48 induces EBL cell apoptosis [66], which is in contrast to GcvH regulation on EBL cell apoptosis in our study. This suggests that distinct *M bovis* conserved proteins have different or even opposite effects on EBL cell apoptosis. This phenomenon is not uncommon among pathogens. For instance, in *Mycobacterium tuberculosis*, the conserved lipoprotein LpqT inhibits apoptosis in mouse bone marrow-derived macrophages (BMDMs) while the MPT83 induces apoptosis in this cell line [67,68], and in Hepatitis B virus, the conserved protein HBX triggers apoptosis in a human hepatoma cell line (HeGp2) while HBC inhibits apoptosis in this cell line [69,70]. We hypothesized that differences in dominant proteins (anti-apoptotic or pro-apoptotic proteins) at distinct infection stages or in various strains may determine how *M*. *bovis* regulates apoptosis. Mining of the *M*. *bovis* apoptosis-regulating proteins could help elucidate the mechanism by which *M*. *bovis* differentially regulates host cell apoptosis.

Apoptosis is typically triggered by extrinsic (death receptor) and intrinsic (mitochondrial or endoplasmic reticulum (ER)-mediated) apoptotic pathways. Infections frequently target the intrinsic apoptotic pathway to avoid or counteract host cell apoptosis. Poxviruses, for example, encode the M1 protein to block mitochondria-mediated intrinsic apoptotic pathway, and *Anaplasma phagocytophilum* secrete Ats-1 proteins that localize to host mitochondria to prevent mitochondria-mediated intrinsic apoptotic pathway activation [71,72]. *Brucella abortus* VceC suppresses ER-mediated intrinsic apoptotic pathway in goat trophoblast cells [73]. In this study, we found that both intrinsic apoptotic pathways are involved in GcvH anti-host cell apoptosis. GcvH employs an interesting strategy to hijack both intrinsic apoptotic pathways by interfering with ER-associated apoptotic signaling, then transmitting it to the mitochondria via CHOP and eventually to the Caspase 3. This was further supported by the subsequent screening out of Brsk2, a host protein that localizes to the ER and interacts with GcvH.

Brsk2, an AMP-activated protein kinase (AMPK)-associated kinase, is abundant in the pancreas, frontal cortex and brain [74,75]. Although Brsk2 has been mainly studied for its function in neuronal polarization, cell cycle progression and insulin production, its critical role in the ER-associated apoptotic pathway has also been noted [76,77]. Previous studies showed that Brsk2 knockdown increases CHOP transcription and apoptotic levels, while its overexpression decreases them [77,78]. Consistent with these findings, we discovered that GcvH interacting with Brsk2 enhances Brsk2 expression to decrease CHOP expression, which in turn inhibits Caspase-3 cleavage activation and apoptosis. We further demonstrated that GcvH does not affect Brsk2 protein synthesis, but rather increases its stability to mediate an increase in Brsk2 protein levels, thus inhibiting CHOP and host cell apoptosis. Furthermore, we determined the key amino acid region for the interaction between GcvH and Brsk2. On the one hand, a mutation of this key region confirmed that this interaction is required for GcvH to increase Brsk2 level and resist host cells apoptosis; on the other hand, multiple sequence comparisons on representative mycoplasmas containing the GCS showed that this region is very highly conserved, suggesting the interaction with Brsk2 may be conserved.

Interactions with host molecules determine bacterial adherence and entry into host cells as well as its cellular and tissue tropism [79,80]. *M*. *bovis* can invade the ears, lungs, lymph nodes, brain and heart of cattle [81]. Mhp was isolated from the livers, spleens, kidneys, and bronchial lymph nodes of experimentally infected swine [82,83]. Clearly, the ability of animal mycoplasmas to invade a wide range of host tissues is being gradually recognized, although the detail of the mechanism needs further study. Interactions with many different cellular proteins may explain the broad tissue tropism seen in animal mycoplasma infections [5]. In our study, we confirmed Brsk2 as an interaction target of ruminant and swine mycoplasmas, underlining the possibility of their broad tissue tropism, given that Brsk2 is abundant in the pancreas, frontal cortex and brain.

In conclusion, we propose a model of the molecular mechanism underlying GCS-containing mycoplasmas anti-host cell apoptosis. This study unveils strategies by which reduced-genome bacteria exploit a limited number of genomic proteins to resist host cell apoptosis thereby causing disease. Understanding the mechanisms governing the ability of mycoplasmas to manipulate the immune response is valuable for the development of therapeutic and prophylactic interventions targeting the host. This is of paramount importance to control mycoplasmas that insidiously hijack and deregulate innate immune anti-bacterial responses to facilitate mycoplasma propagation and infection.

## Materials and methods

### Mycoplasma strains, cells, and culture conditions

*M*. *bovis* TJ strain was isolated from the lung of a diseased cattle with pneumonia in Tianjin, China. Strain TJ was grown in PPLO medium (Basal Media, Shanghai, China) and used at low passage number (< 10). To estimate the number of colony-forming unit (CFU) in the cultures, serial dilutions were plated on a modified PPLO medium containing 1.5% agarose (V2111; Promega) and incubated at 37°C. CFU was counted 7–10  days later using a microscope [84].

Embryonic bovine lung epithelial (EBL) cells were kindly provided by Prof. Fei Xue of State Key Laboratory for Animal Disease Control and Prevention, Harbin Veterinary Research Institute, Chinese Academy of Agricultural Sciences, Harbin, China. HeLa cells were purchased from the American Type Culture Collection (ATCC). Porcine tracheal epithelial (PTE) cells were prepared from the tracheas of two 5-week-old specific-pathogen-free (SPF) piglets using previously described protocols [85]. Goat tracheal epithelial (GTE) cells were kindly provided by Prof. Maojun Liu of Key Laboratory for Veterinary Bio-Product Engineering, Ministry of Agriculture and Rural Affairs, Jiangsu Academy of Agricultural Sciences, Nanjing, China. These cell lines were cultured in Dulbecco’s modified Eagle’s medium (DMEM) supplemented with 10% heat-inactivated fetal bovine serum (FBS), 100 μg/ml streptomycin (Gibco), 100 U/ml penicillin (Gibco), and 10 mM HEPES (Invitrogen). The cells were incubated at 37°C in 5% CO_2_.

For infection experiments, EBL cells were maintained in DMEM supplemented with 10% FBS, 100 U/ml penicillin, and 100 μg/ml streptomycin, inoculated with *M*. *bovis* cells at a multiplicity of infection (MOI) of 50.

### MAPs preparation and grouping

*M*. *bovis* MAPs were prepared as described previously [86]. Briefly, *M*. *bovis* was cultivated until the beginning of the stationary growth phase (when a red pH indicator turned orange) and then collected by centrifugation. *M*. *bovis* cells were washed with PBS via pipetting up/down and briefly vortexing, and then harvested by centrifugation at 8000 × g for 30 min at 4°C. The pellets resuspended in 5 ml of Tris-buffered saline (TBS; 50 mM Tris-Cl, pH 8.0, 0.15 M NaCl) containing 1 mM EDTA (TBSE), to which Triton X-114 was added to a final concentration of 2% and incubated at 4°C for 1 h. The lysate was then incubated at 37°C for 10 min for phase separation. After centrifugation, the upper aqueous phase was removed and replaced with the same volume of TBSE. The solution was vortexed and incubated at 4°C for 10 min. The phase separation process was repeated twice. The final Triton X-114 phase was resuspended in TBSE to the original volume, and 2.5-fold volumes of ethanol were then added to precipitate the membrane components overnight at -20°C. After centrifugation, the pellet was resuspended in PBS and lysed by sonication. Protein concentrations were examined using the Bradford assay (Thermo Scientific, Waltham, MA, USA). The endotoxin concentration of the heat-inactivated mycoplasma MAPs was <0.04 endotoxin units/ml, as checked by the Limulus amebocyte lysate assay (Associates of Cape Cod, Falmouth, MA, USA). *M*. *bovis* MAPs were loaded onto a HiLoad 16/600 Superdex 200 pg prepacked column (GE Health Care) and separated using loading buffer (100 mM NaCl, 20 mM KCl and 20 mM Tris-HCl pH 8.5) on AKTA avant system (GE Health care). Proteins were harvested by UV absorbance at 280 and then resuspended in PBS after ultrafiltration.

### Protein expression and purification

The nucleotide sequence encoding the *M*. *bovis* TJ strain GcvH (WP_013954496.1) was codon-optimized and synthesized by Genesoul Technology (Harbin, China). The sequence was cloned into the pMAL-c6T (New England Biolabs, N-terminal 6×His-MBP) vector with the restriction enzymes *Sal* I and *Bam* HI. The recombinant plasmid was transformed into *E*. *coli* BL21(DE3) cells. The level of recombinant protein expression was analyzed by SDS-PAGE. Subsequently, the GcvH-MBP fusion proteins were purified by using amylose agarose resin (New England Biolabs) according to the manufacturer’s instructions. The amylose elution was followed by incubation with Tobacco Etch Virus nuclear-inclusion-a endopeptidase (TEV) Protease at 4°C overnight, in a buffer containing 20 mM Tris-HCl pH 7.5, 200 mM NaCl, and 10 mM imidazole. Free MBP and TEV protease, both containing a His-tag, were removed on a Ni-NTA affinity column (GE Healthcare). GcvH protein was concentrated and resuspended in PBS using a 3 kDa ultrafiltration tube (Millipore). GcvH protein purification was analyzed by SDS-PAGE, and the protein concentration was determined with a BCA Protein Quantitation Kit (Beyotime, China).

### Purifying antibodies against *M*. *bovis* GcvH

Anti-*M*. *bovis* GcvH antibodies were purified from the murine serum anti-GcvH as described previously [87]. Three milliliters of antiserum were mixed with 6 ml of 60 mM sodium acetate (pH 4.0), and 100% caprylic acid (for a final concentration of 2.5%) was added while the mixture was stirred. Upon stirring for 20–30 min, the mixture was centrifuged at 5100 g for 20 min, and the supernatant was collected (Total IgG). One milliliter amylose resin was poured into a 5 ml empty column and washed with five column volumes of Column Buffer (20 mM Tris–HCl, 200 mM NaCl and 1 mM EDTA). The resin was incubated with 5 mg GcvH-MBP protein for 30 min at room temperature. After washing the column with three column volumes of Column Buffer, the total IgG was passed through the column three times. About 3.5 M potassium thiocyanate was directly added onto the beads to elute the antibody, prior to which the columns were rinsed with 10 volumes of Column Buffer and 0.5 M NaCl-PBS, respectively. Two-column volumes of 10 mM sodium bicarbonate were passed through a desalting column and the eluted antibody was loaded onto the column. The column was placed in the 50 ml centrifuge tube and centrifuged at 2000 rpm for 5 min. The liquid (purified antibody) at the bottom of the centrifuge tube was collected and the IgG concentration was determined by UV spectroscopy (275 nm).

### Blocking assay

*M*. *bovis* were washed thrice with PBS and preincubated with the purified antibodies against GcvH (10, 20 and 50 μg/ml) or PBS at 37°C for 30 min. These bacteria were washed and then added to EBL cells (MOI = 50). Following incubation for 12 h, the cells were washed three times with PBS and subjected to Western blotting analysis.

### Plasmids or siRNAs construction and transfection

The nucleotide sequences encoding the GcvH from *M*. *bovis* TJ strain (WP_013954496.1), *M*. *capricolum subspecies capripneumoniae* (Mccp) reference strain F38 (WP_019269436.1), and *M*. *hyopneumoniae* (Mhp) 232 strain (WP_014579758.1) were codon-optimized and synthesized into the pCAGGS-Flag or pEGFP-C1 vector by Genesoul Technology (Harbin, China). Three truncated GcvH constructs based on the plasmid pCAGGS-Flag (express the aa 1–38, 39–76, and 77–114 of GcvH and named GcvH-Flag-P1, GcvH-Flag-P2 and GcvH-Flag-P3, respectively), and the deleted constructs based on the plasmid pEGFP-GcvH-P1 (with the aa 1–7, 8–14, 15–22, 23–30, 31–38, 31–32, 33–35 or 36–38 deleted) were also synthesized by Genesoul Technology.

The bovine Brsk2 gene (XM_024987601.2) was codon-optimized and synthesized into pCAGGS-Myc by the Beijing Genomics Institute (BGI, Beijing, China). The mutant plasmid (GcvH-Flag I31A+G32A), with isoleucine (I) at position 31 and glycine (G) at position 32 both mutated to alanine (A), were constructed in GcvH-Flag plasmid using Mut Express MultiS Fast Mutagenesis Kit (Vazyme, China).

The siBrsk2-1 (sense strand: 5’-GCG AGU CUG UAC UGA UGA ATT-3’ and antisense strand: 5’-UUC AUC AGU ACA GAC UCG CTT-3’), siBrsk2-2 (sense strand: 5’-GCA CAU UCA GAA ACA CAU ATT-3’ and antisense strand: 5’-UAU GUG UUU CUG AAU GUG CTT-3’) and siBrsk2-3 (sense strand: 5’-GGC UCA ACU CCA UCA AGA ATT-3’, and antisense strand: 5’-UUC UUG AUG GAG UUG AGC CTT-3’) were synthesized by GenePharma (Shanghai, China).

PolyJet DNA in vitro transfection reagent (SignaGen, USA) was used to transfect recombinant plasmids into EBL or Hela cells, and jetPRIME transfection (Polyplus, USA) was used to transfect siRNAs into EBL cells.

### Cell viability measurements

Cell viability was detected using a Cell Counting Kit-8 (CCK-8) according to the manufacturer’s protocol (Vazyme, Nanjing, China). CCK-8 measures cell viability by correlating the production of colored formazan dye to the number of living cells in the culture.

### Western blotting

Whole-cell lysates were harvested at the indicated time points. An equal number of cells were lysed with the cell lysis buffer for 30 min at 4°C. The protein concentration was determined using a BCA Protein Assay Kit (Beyotime, Shanghai, China). Equal amounts of total cell lysates were separated by SDS-PAGE. The proteins in the gel were transferred onto nitrocellulose membranes (Pall Corporation, USA), which were then blocked with 5% cold-water fish skin gelatin in TBST (Solarbio, Beijing, China) at 4°C overnight and then incubated for 2 h with different primary antibodies at room temperature (RT). A summary of antibodies used in this study is listed in S1 Table. After washing with TBST, DyLight 800-labeled goat anti-mouse or anti-rabbit IgG (H + L) (1:10,000, Kirkegaard & Perry Laboratories, Gaithersburg, USA) was used for detection. The membrane was scanned using an Odyssey infrared imaging system, and the fluorescence intensity of each band was measured using Odyssey 2.1 software (LI-COR Biosciences).

### Terminal deoxynucleotidyl transferase-mediated dUTP-biotin nick end labeling (TUNEL) assay

Apoptotic cells were examined using a TUNEL BrightGreen Apoptosis Detection Kit (Vazyme; A112-01) according to the manufacturer’s instructions. Briefly, EBL cells were seeded into 48-well plates. After incubation with GcvH and/or Staurosporine (STS, Beyotime, #S1882), the cells were fixed with 4% paraformaldehyde (PFA) for 30 min at RT. After rinsing thrice with PBS, the cells were permeabilized using freshly prepared 0.2% Triton X-100 for 5 min. The cells were then overlaid with 100 μl of 1×Equilibration Buffer and incubated for 30 min at RT. TdT buffer was added to EBL cells and incubated for 1 h at 37°C after discarding the Equilibration Buffer. Finally, the cells were rinsed thrice with PBS and stained with DAPI (4’, 6-diamidino-2-phenylindole) (0.05 μg/ml, Sigma) at RT for 15 min in the dark and directly analyzed under a fluorescence microscope (EVOS M5000, Invitrogen).

### Flow cytometric analysis of apoptosis

EBL cells were seeded into six-well plates and mock-infected or infected with *M*. *bovis* (MOI = 50) for 12 or 24 h. EBL cell apoptosis was determined using a FITC Annexin V Apoptosis Detection Kit (BD Pharmingen). According to the manufacturer’s manual, cells were harvested by centrifugation at 1,000 g for 5 min, rinsed thrice with PBS, and resuspended in 100 μl of 1 × binding buffer. The cells were then incubated with FITC-conjugated Annexin V and propidium iodide at RT for 15 min in the dark. 1 × binding buffer (400 μl) was then added to the mixture and the percentage of apoptotic cells was determined by flow cytometry (A60-Universal, Apogee, Britain).

### Immunofluorescence

EBL cells were incubated with STS or transfected with vectors (PCAGGS-Flag, GcvH-Flag or GcvH-Flag I31A+G32A) and then infected with *M*. *bovis* at an MOI of 50. EBL cells were rinsed thrice with PBS and fixed in 4% PFA for 30 min. The fixed cells were permeabilized with 0.1% Triton X-100 for 15 min and blocked for 1 h in 5% cold-water fish skin gelatin. Cells were then incubated with mouse anti-*M*. *bovis* GAPDH protein monoclonal antibodies AD10 (produced in our laboratory) for 1 h at RT, washed thrice with PBS and incubated with secondary antibodies coupled to Alexa Fluor 488 (ThermoFisher) for 1 h. The cell nuclei were labeled with DAPI and the cell number was counted using ImageJ. Fluorescence images were acquired using a fluorescence microscope (EVOS M5000, Invitrogen).

### Real-time quantitative RT-PCR and TaqMan probe assay

Total RNA was extracted from the cells using a RNeasy Mini kit (Qiagen Sciences, Hilden, Germany) according to the manufacturer’s instructions. RNA was reverse transcribed using a Transcriptor First-Strand cDNA Synthesis Kit (Roche Diagnostics, Indianapolis, USA). qPCR was performed in triplicate using FastStart Universal SYBR Green Master Mix (Rox) (Roche Diagnostics, Indianapolis, USA). All data were acquired using a QuantStudio 5 real-time PCR system (Applied Biosystems, Carlsbad, USA). The expression value of each gene was normalized to that of GAPDH, and final values were calculated using the ΔΔCt method. The results were analyzed using QuantStudio Design & Analysis software v1.4 (Applied Biosystems). The ATF4 and XBP1s primer sequences used in this study are provided in S2 Table.

The level of cell-associated mycoplasmas in *M*. *bovis*-infected EBL cells were determined by using a specific TaqMan probe assay [88]: the forward primer (F: 5’-GAGAATGCTTCAGTATTTTGACGG-3’), reverse primer (R: 5’-CAAAAGCAAAATGTTAAATTCAGG-3’), and TaqMan probe (5’-FAM-CATATATAAGTGAGACTAACTTATT-MGB-3’). Briefly, EBL cells in pairs of two wells were washed twice with PBS following exposure to *M*. *bovis*. EBL cells in one well were collected using trypsin to count numbers with a Neubauer counting chamber, while EBL cells in the other well along with *M*. *bovis* were harvested using a cell scraper. The genomic DNA of cell-associated *M*. *bovis* was extracted and its copy number was quantitated by TaqMan qPCR. The composition of the TaqMan qPCR assay mixture was according to the manufacturer’s protocol (Premix Ex Taq (Probe qPCR), TaKaRa, China). Amplification was performed using a QuantStudio 5 real-time PCR system. In each plate, 10-fold dilutions of the standard plasmid (10^11^−10^5^ copies/μl) and the blank control were included. Each sample was assayed three times.

### Mitochondrial membrane potential detection

To detect the mitochondrial membrane potential of EBL cells, the mitochondrial probe JC-1 was used according to the manufacturer’s instructions (Beyotime, C2006). EBL cells were seeded into the glass bottom CellCulture Dish (Nest, China) and then incubated with 0, 0.25, 0.50 or 1.00 μg/ml GcvH protein. The mitochondrial electron transport chain inhibitor CCCP (50 μM) provided in the kit was used as a positive control and blank cells were used as a negative control. After discarding the cell culture medium, EBL cells were fixed with 4% PFA for 30 min at RT and washed 3 thrice with PBS. 0.5 ml DMEM complete medium and 0.5 ml JC-1 working solution were added and incubated at 37°C for 20 min. The cells were washed thrice with 1 ml JC-1 staining buffer (1×) and resuspended in 1 ml of DMEM complete medium to observe the amount of red and green fluorescence by the laser confocal microscopy (Zeiss, LSM980).

### GST pull-down assay

EBL cells were harvested and resuspended in 2 ml of RIPA lysate containing protease inhibitor. The cells were lysed on ice for 1 h and centrifuged at 12000 r/min at 4°C for 15 min to collect the supernatant. 200 μl glutathione 4B-Sepharose beads (GE Biosciences) were added to the affinity chromatography column and washed with PBS twice. GST or the GST-GcvH protein produced in *E*. *coli* BL21(DE3) cells was added in the affinity chromatography column to conjugate to glutathione-agarose beads. The beads were then washed with PBS thrice and incubated with the supernatant from the lysed EBL cells for 2 h at RT. After five washes with lysis buffer, the protein complexes binding to the beads were eluted with 75 μl of 1 × GSH elution buffer. Eluted proteins were separated by SDS-PAGE and silver-stained (Beyotime, Nantong, China). The excised gel band was sent to BGI (Beijing, China) and analyzed by mass spectrometry.

### Co-IP assays

The plasmid expressing Brsk2-Myc protein was cotransfected with the plasmids expressing *M*. *bovis* proteins (GcvH, GcvH truncates, or GcvH mutations) into Hela cells, respectively. At 36 h posttransfection, the cells were lysed with ice-cold lysis buffer for 30 min at 4°C. After centrifugation (8000g, 10 min) at 4°C, the clarified extracts were incubated with magnetic beads conjugated with anti-Flag or anti-Myc antibody (Sigma-Aldrich, USA) on a MACSmix Tube rotator at 4°C overnight. The incubated beads were washed five times with lysis buffer, followed by SDS-PAGE and Western blotting.

### Confocal microscopy

To check the subcellular localization of prokaryotically expressed GcvH and *M*. *bovis* membrane component GcvH. EBL cells were transfected with pDsRed2-ER (Clontech, USA) for 24 h and then incubated with 1 μg/ml GcvH protein or *M*. *bovis* (MOI = 50) for 12 h. Extracellular mycoplasmas were killed by an addition of 400 μg/ml gentamicin sulfate (Sigma-Aldrich, #G1914) for an additional 3 h. To observe the co-localization of GcvH and Brsk2, Hela cells were transfected with GcvH-Flag and/or Brsk2-Myc.

These cells were fixed with 4% PFA in PBS for 30 min at RT. After permeabilizing with 0.1% Triton X-100 for 15 min, the cells were blocked for 1 h in 5% cold-water fish skin gelatin. The cells were then incubated with the purified specific mouse anti-GcvH, rabbit anti-Flag or mouse anti-Myc antibodies for 1 h at RT, washed thrice with PBS, and incubated with secondary antibodies coupled to Alexa Fluor 633 or Alexa Fluor 488 (ThermoFisher) for 1 h. The cell nuclei were labeled with DAPI. Fluorescence was observed by confocal microscopy (LSM980, Zeiss).

### Statistical analysis

GraphPad Prism software (version 9.0; GraphPad Software Inc.) was used for all statistical analyses. Data obtained from several experiments are reported as the mean ± SD. The significance of differences between the two groups was determined with a two-tailed Student’s *t* test. One-way analysis of variances (ANOVA) with Dunnett’s or Tukey’s test was employed for multigroup comparisons. For all analyses, a probability (p) value of < 0.05 was considered statistically significant.

## Supporting information

S1 Fig (A) Mock- and *M*. *bovis*-infected EBL cells were labeled with TUNEL (green) and subsequently stained with DAPI, and photomicrographs of TUNEL labeling in *M*. *bovis*-infected cells were obtained using a fluorescence microscope. (B) The pre-infection of *M*. *bovis* diminished the staurosporine (STS)-induced EBL cell apoptosis. (C-D) EBL cells were pretreated with 0.1 or 0.2 μM STS for 2 h, and subjected to detect the cleaved Caspase-3 level by Western blotting (C); or to incubate with *M*. *bovis* for 12 or 24 h, and an IFA was performed to test the number of mycoplasmas per EBL cells. DAPI was used to stain cellular nuclei (blue) (D). All assays were performed with three independent experiments, and values represent the means ± SDs. Significance was assessed by one-way ANOVA with Tukey’s multiple comparison test. ****, *p < 0*.*0001*.
(TIF)

S2 Fig (A-B) *M*. *bovis* MAPs were extracted and then separated into five groups using AKTA. molecular sieves. (C) The group-D MAPs had no effects on EBL cell viability. (D) Mass spectrometry (MS) analysis revealed matches to four peptides of GcvH with approximately 35% sequence coverage. (E) The amino acid sequence of GcvH is shown and matching peptides detected in MS analysis was highlighted in red. (F) The cleaved Caspase-3 levels in EBL cells incubated with several candidate proteins (variable surface lipoprotein (VSPHB0801-4), pyruvate dehydrogenase (PDH), DJ-1, elongation factor Tu (EF-TU), glyceraldehyde 3-phosphate dehydrogenase (GAPDH), and GcvH) were determined by Western blotting. (G-H) The purified GcvH-MBP and GcvH protein were identified by SDS-PAGE (the arrowhead). (I) GcvH protein had no effect on the cell viability of EBL cells. (J) GcvH preincubation attenuate the cytotoxic effects of STS on EBL cells. (K) CCK-8 assays showed that GcvH alleviated the detrimental impact of Tu on EBL cell viability. The data were normalized to the corresponding values in control cells and represented the means ± SDs of the results from three independent experiments. Significance was assessed by one-way ANOVA with Dunnett’s multiple comparison tests relative to the control (I) or with Tukey’s multiple comparison test (J and K). *, *p < 0*.*05*; **, *p < 0*.*01*; ****, *p < 0*.*000*1.
(TIF)

S3 Fig (A-B) qPCR analysis of the ATF4 and XBP1s mRNA levels in EBL cells incubated with GcvH. (C) EBL cells were infected with *M*. *bovis* (MOI = 50) for 12 h, and then intracellular *M*. *bovis* membrane protein GcvH (green) was localized using the purified specific anti-GcvH antibody conjugated with an anti-mouse fluorescent secondary antibody. The EBL cell’s ER (red) was labeled by transfecting with the plasmid pDsRed2-ER. DAPI was used to stain cellular nuclei (blue). Quantifications were normalized to those of the control, and data are presented as the means ± SDs from three independent experiments, and significance was assessed by one-way ANOVA with Dunnett’s multiple comparison test relative to the control. *, *p < 0*.*05*; **, *p < 0*.*01*; ***, *p < 0*.*001*; ****, *p < 0*.*0001*.
(TIF)

S4 Fig (A) GST and GST-GcvH proteins were prokaryotic expression and purification. (B) EBL cells were transfected with siRNAs (siBrsk2-1, -2 and -3) for 48 h and harvested for Western blotting analysis to verify the efficiency of Brsk2 knockdown. siRNA-NC represents the control siRNA. (C) EBL cells were transfected with pCAGGs-Flag or GcvH-Flag for 48 h and harvested for qPCR to detect Brsk2 mRNA level. (D) EBL cells coexpressing Brsk2-Myc and GcvH-Flag or empty vector control (Flag) were treated with the proteasomal inhibitor MG-132 (10 μM, 12 h). Brsk2 and β-actin levels were determined by Western blotting analysis. (E) GcvH did not promote *M*. *bovis* infection of EBL cells when it lost the interaction with Brsk2. EBL cells were transfected by pCAGGS-Flag, GcvH-Flag or GcvH-Flag mutant plasmid, and then subjected to detect the number of mycoplasmas per EBL cells by a TaqMan qPCR analysis. The data were normalized to the control (transfection with pCAGGS-Flag), and data are presented as the means ± SDs from three independent experiments, and significance was assessed by a two-tailed Student’s *t* test (C) or one-way ANOVA with Tukey’s multiple comparison test (E). ****, *p < 0*.*0001*.
(TIF)

S1 Table Antibodies used in this study. (DOCX)

S2 Table qPCR primers used in the experiment. (DOCX)

S1 Data Source data for graphs in this study. (XLSX)

S2 Data The raw data of western blot. (PDF)
