# Supplementary figures and images for: Mycoplasma glycine cleavage system key subunit GcvH is an apoptosis inhibitor targeting host endoplasmic reticulum
Source: PLoS Pathog. 2024 May 24;20(5):e1012266. doi: 10.1371/journal.ppat.1012266 (PMC11156438; doi:10.1371/journal.ppat.1012266)

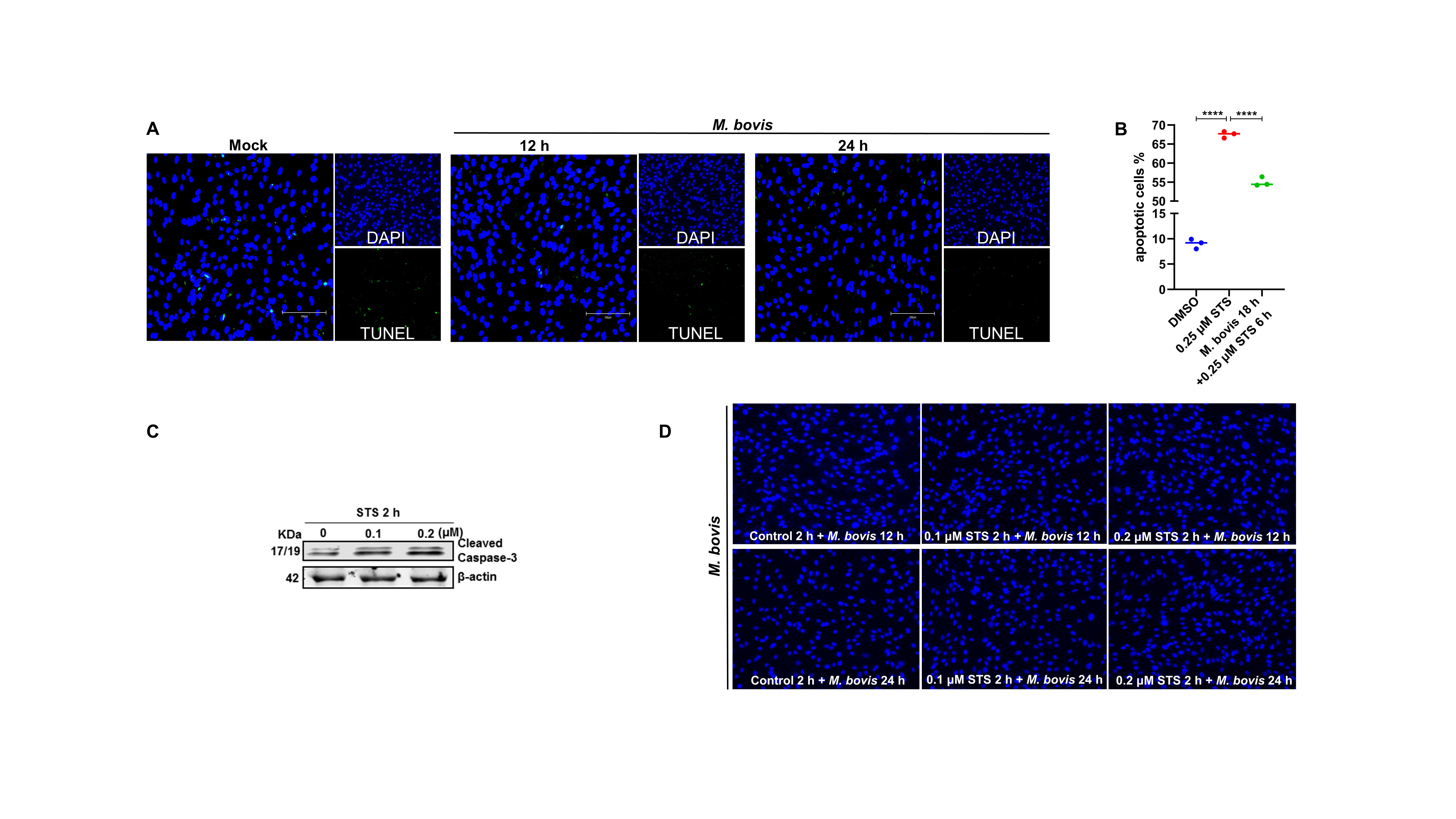

Supplement: S1 Fig — (A) Mock- and M. bovis-infected EBL cells were labeled with TUNEL (green) and subsequently stained with DAPI, and photomicrographs of TUNEL labeling in M. bovis-infected cells were obtained using a fluorescence microscope. (B) The pre-infection of M. bovis diminished the staurosporine (STS)-induced EBL cell apoptosis. (C-D) EBL cells were pretreated with 0.1 or 0.2 μM STS for 2 h, and subjected to detect the cleaved Caspase-3 level by Western blotting (C); or to incubate with M. bovis for 12 or 24 h, and an IFA was performed to test the number of mycoplasmas per EBL cells. DAPI was used to stain cellular nuclei (blue) (D). All assays were performed with three independent experiments, and values represent the means ± SDs. Significance was assessed by one-way ANOVA with Tukey’s multiple comparison test. ****, p < 0.0001. (TIF) [file ppat.1012266.s001.tif]

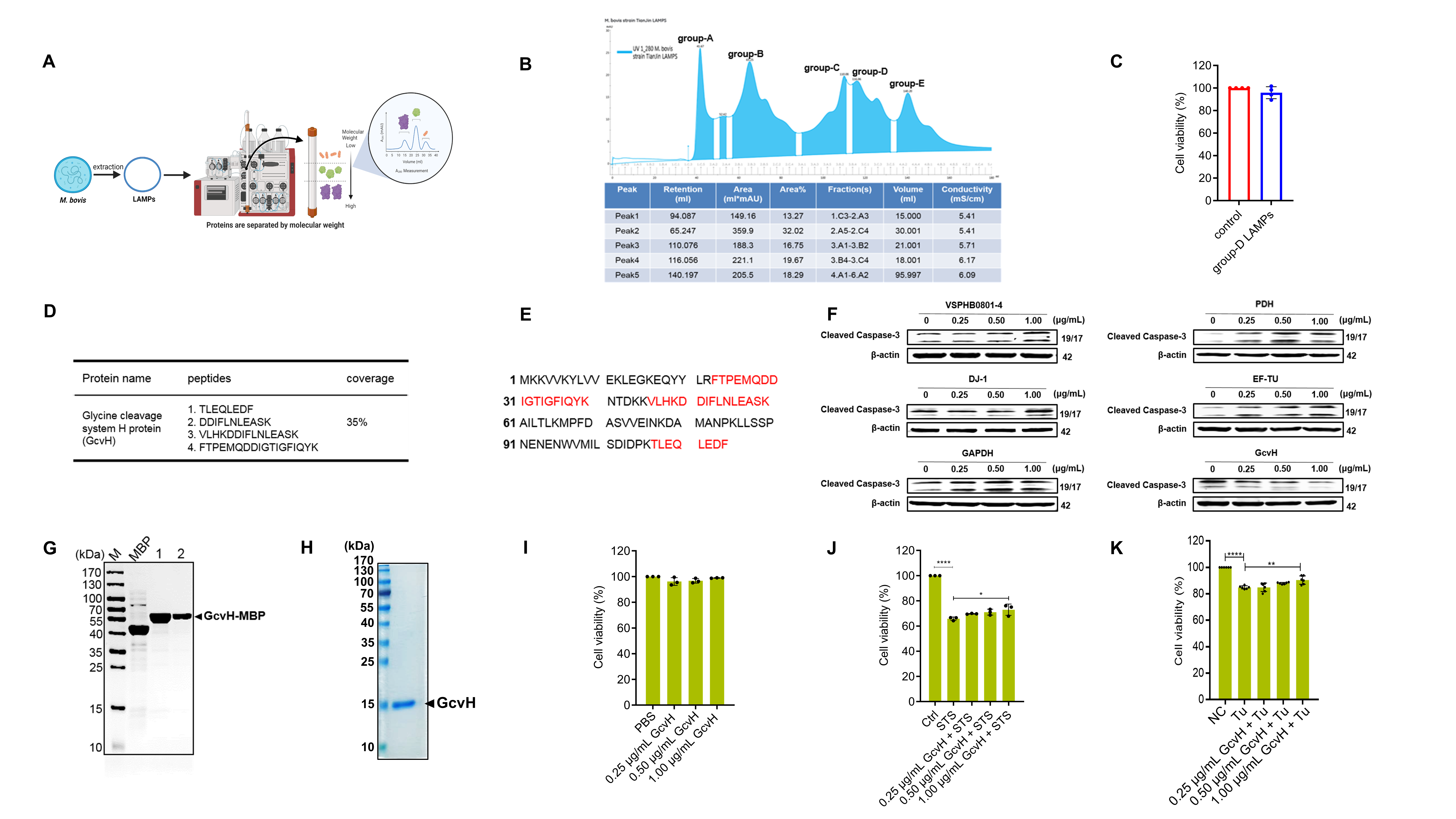

Supplement: S2 Fig — (A-B) M. bovis MAPs were extracted and then separated into five groups using AKTA. molecular sieves. (C) The group-D MAPs had no effects on EBL cell viability. (D) Mass spectrometry (MS) analysis revealed matches to four peptides of GcvH with approximately 35% sequence coverage. (E) The amino acid sequence of GcvH is shown and matching peptides detected in MS analysis was highlighted in red. (F) The cleaved Caspase-3 levels in EBL cells incubated with several candidate proteins (variable surface lipoprotein (VSPHB0801-4), pyruvate dehydrogenase (PDH), DJ-1, elongation factor Tu (EF-TU), glyceraldehyde 3-phosphate dehydrogenase (GAPDH), and GcvH) were determined by Western blotting. (G-H) The purified GcvH-MBP and GcvH protein were identified by SDS-PAGE (the arrowhead). (I) GcvH protein had no effect on the cell viability of EBL cells. (J) GcvH preincubation attenuate the cytotoxic effects of STS on EBL cells. (K) CCK-8 assays showed that GcvH alleviated the detrimental impact of Tu on EBL cell viability. The data were normalized to the corresponding values in control cells and represented the means ± SDs of the results from three independent experiments. Significance was assessed by one-way ANOVA with Dunnett’s multiple comparison tests relative to the control (I) or with Tukey’s multiple comparison test (J and K). *, p < 0.05; **, p < 0.01; ****, p < 0.0001. (TIF) [file ppat.1012266.s002.tif]

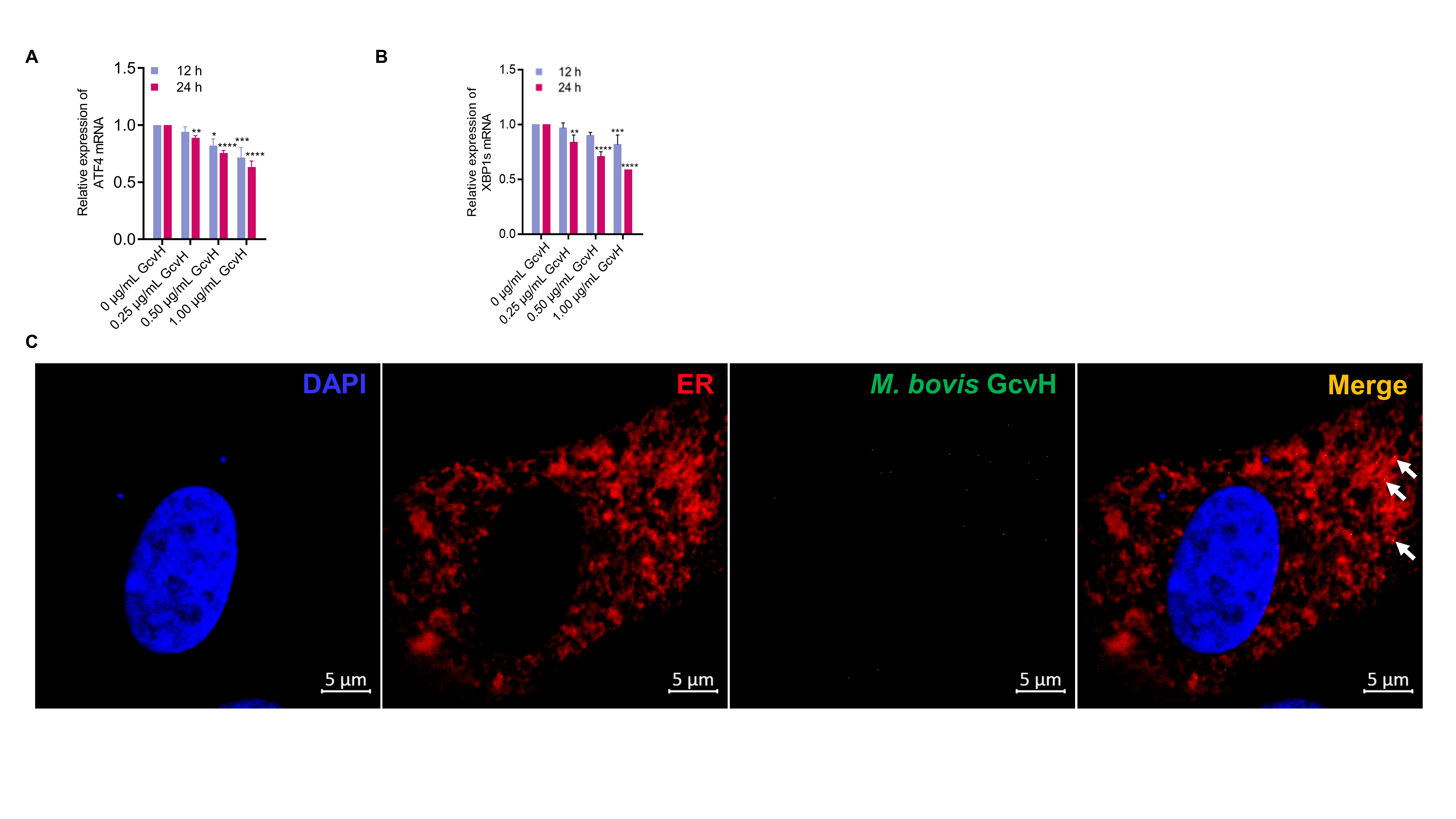

Supplement: S3 Fig — (A-B) qPCR analysis of the ATF4 and XBP1s mRNA levels in EBL cells incubated with GcvH. (C) EBL cells were infected with M. bovis (MOI = 50) for 12 h, and then intracellular M. bovis membrane protein GcvH (green) was localized using the purified specific anti-GcvH antibody conjugated with an anti-mouse fluorescent secondary antibody. The EBL cell’s ER (red) was labeled by transfecting with the plasmid pDsRed2-ER. DAPI was used to stain cellular nuclei (blue). Quantifications were normalized to those of the control, and data are presented as the means ± SDs from three independent experiments, and significance was assessed by one-way ANOVA with Dunnett’s multiple comparison test relative to the control. *, p < 0.05; **, p < 0.01; ***, p < 0.001; ****, p < 0.0001. (TIF) [file ppat.1012266.s003.tif]

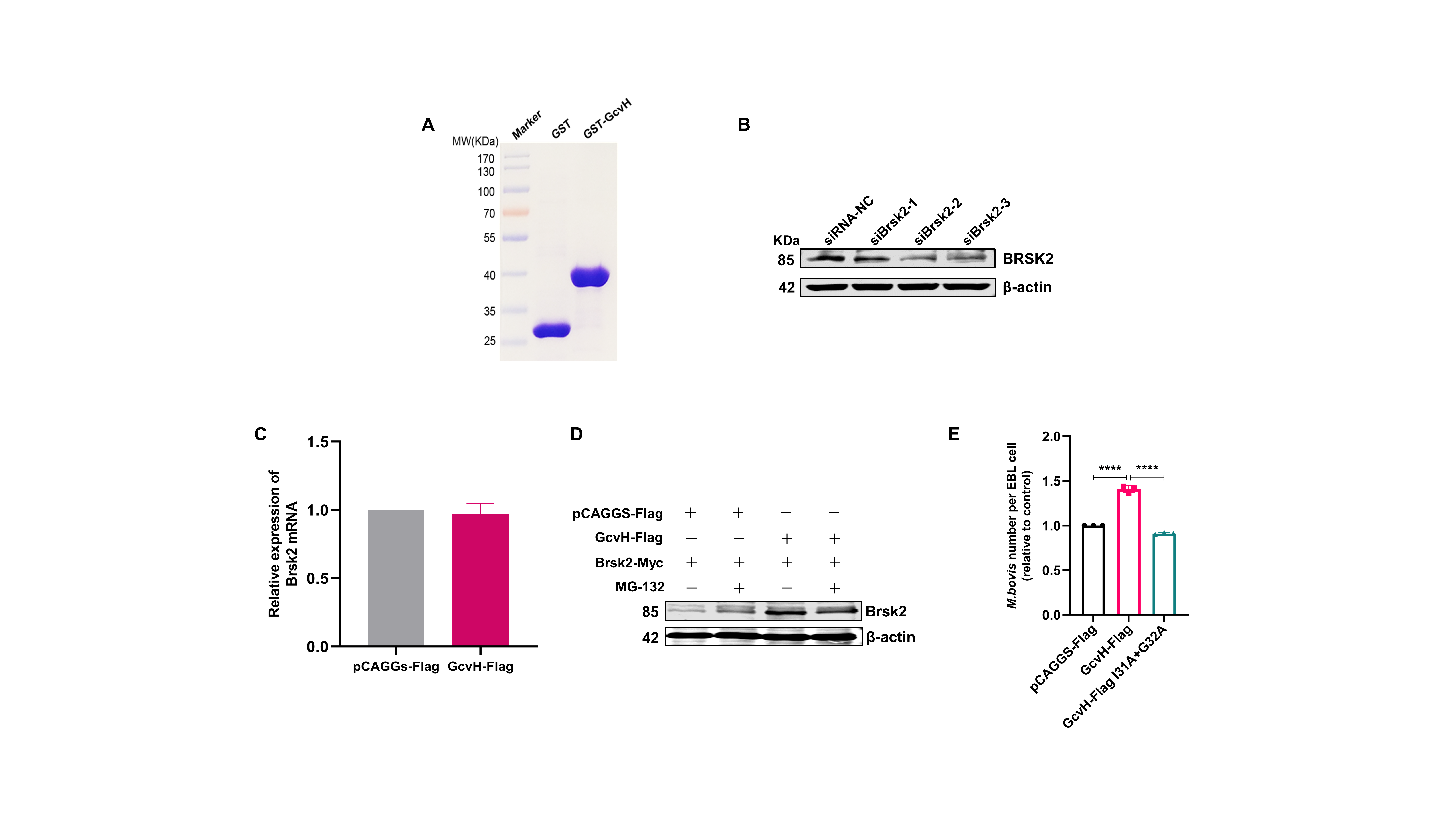

Supplement: S4 Fig — (A) GST and GST-GcvH proteins were prokaryotic expression and purification. (B) EBL cells were transfected with siRNAs (siBrsk2-1, -2 and -3) for 48 h and harvested for Western blotting analysis to verify the efficiency of Brsk2 knockdown. siRNA-NC represents the control siRNA. (C) EBL cells were transfected with pCAGGs-Flag or GcvH-Flag for 48 h and harvested for qPCR to detect Brsk2 mRNA level. (D) EBL cells coexpressing Brsk2-Myc and GcvH-Flag or empty vector control (Flag) were treated with the proteasomal inhibitor MG-132 (10 μM, 12 h). Brsk2 and β-actin levels were determined by Western blotting analysis. (E) GcvH did not promote M. bovis infection of EBL cells when it lost the interaction with Brsk2. EBL cells were transfected by pCAGGS-Flag, GcvH-Flag or GcvH-Flag mutant plasmid, and then subjected to detect the number of mycoplasmas per EBL cells by a TaqMan qPCR analysis. The data were normalized to the control (transfection with pCAGGS-Flag), and data are presented as the means ± SDs from three independent experiments, and significance was assessed by a two-tailed Student’s t test (C) or one-way ANOVA with Tukey’s multiple comparison test (E). ****, p < 0.0001. (TIF) [file ppat.1012266.s004.tif]
