# Supplementary material for: Mycoplasma glycine cleavage system key subunit GcvH is an apoptosis inhibitor targeting host endoplasmic reticulum
Source: PLoS Pathog. 2024 May 24;20(5):e1012266. doi: 10.1371/journal.ppat.1012266 (PMC11156438; doi:10.1371/journal.ppat.1012266)
Supplement: S1 Table — (DOCX) [file ppat.1012266.s005.docx]

| **Antibody** | **Origin** | **Catalog Number** |
| --- | --- | --- |
| **Cleaved PARP1** | Abcam | #ab32064 |
| **Caspase-9** | Abcam | #ab69514 |
| **Caspase-12** | Abcam | #ab62484 |
| **ATF6** | Abcam | #ab37149 |
| **IRE1** | Abcam | #ab48187 |
| **Myc** | Abcam | #ab32 |
| **Caspase-3** | Cell Signaling Technology | #9661S |
| **GRP78** | Cell Signaling Technology | #3177S |
| **CHOP** | Cell Signaling Technology | #2895S |
| **P-JNK** | Cell Signaling Technology | #4668S |
| **Caspase-8** | Huaan Biotechnology | #ET1612-70 |
| **Apaf-1** | Huaan Biotechnology | #ET1607-12 |
| **β-actin** | Zhongshan Goldenbridge-Bio | #TA-09 |
| **PERK** | Affinity Biosciences | #DF7576 |
| **P-eIF2α** | Affinity Biosciences | #AF3087 |
| **ATF4** | Affinity Biosciences | #DF6008 |
| **Bcl-2** | Santa cruz | #sc-7382 |
| **Bax** | Santa cruz | #sc-7480 |
| **Cyt C** | Proteintech | #10993-1-AP |
| **Brsk2** | Proteintech | #11589-1-AP |
| **GFP** | Proteintech | #50430-2-AP |
| **Flag** | Sigma-Aldrich | #F7425 |
