# Supplementary material for: Mycoplasma glycine cleavage system key subunit GcvH is an apoptosis inhibitor targeting host endoplasmic reticulum
Source: PLoS Pathog. 2024 May 24;20(5):e1012266. doi: 10.1371/journal.ppat.1012266 (PMC11156438; doi:10.1371/journal.ppat.1012266)
Supplement: S2 Table — (DOCX) [file ppat.1012266.s006.docx]

**The qPCR primers used in this study.**

| **Primer name** | **Sequence (5’-3’)** |
| --- | --- |
| **ATF4-F** | GGCCAAGCACTTCAAACATC |
| **ATF4-R** | CACCATCCAATCTGTCCCG |
| **XBP1s-F** | TGCTGAGTCCGCAGCAGG |
| **XBP1s-R** | CATCAGAGTCCATGGGGAGA |
| **GAPDH-F** | ATCTCTGCACCTTCTGCCGA |
| **GAPDH-R** | GCAGGAGGCATTGCTGACA |
