# Supplementary material for: Mycoplasma glycine cleavage system key subunit GcvH is an apoptosis inhibitor targeting host endoplasmic reticulum
Source: PLoS Pathog. 2024 May 24;20(5):e1012266. doi: 10.1371/journal.ppat.1012266 (PMC11156438; doi:10.1371/journal.ppat.1012266)

Fig 1B

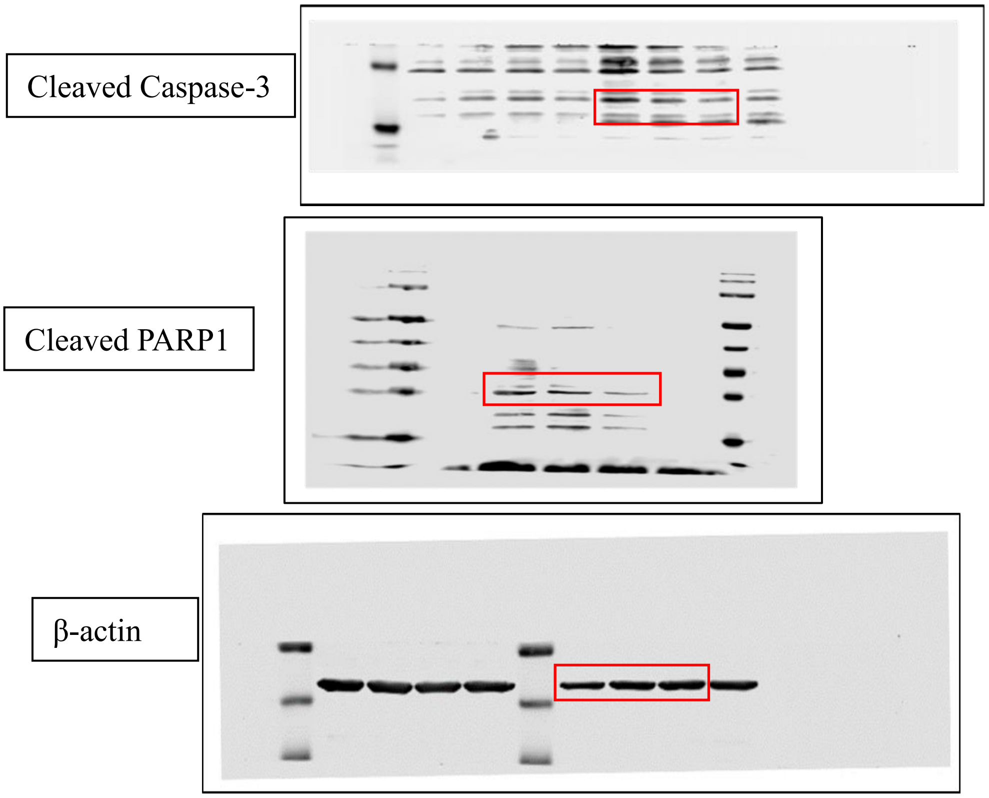

Fig 2C

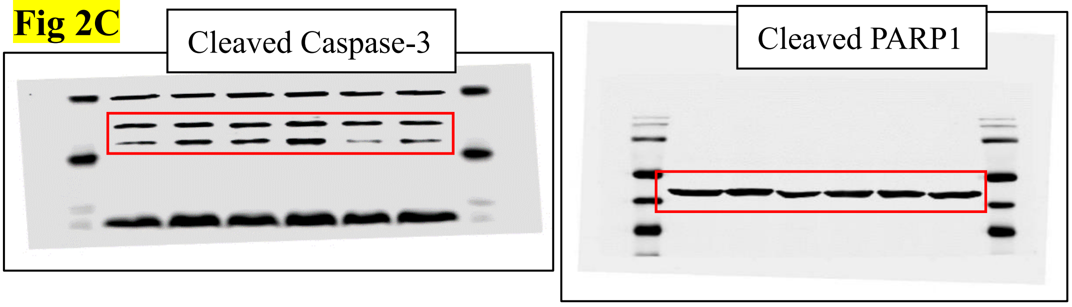

Fig 2D

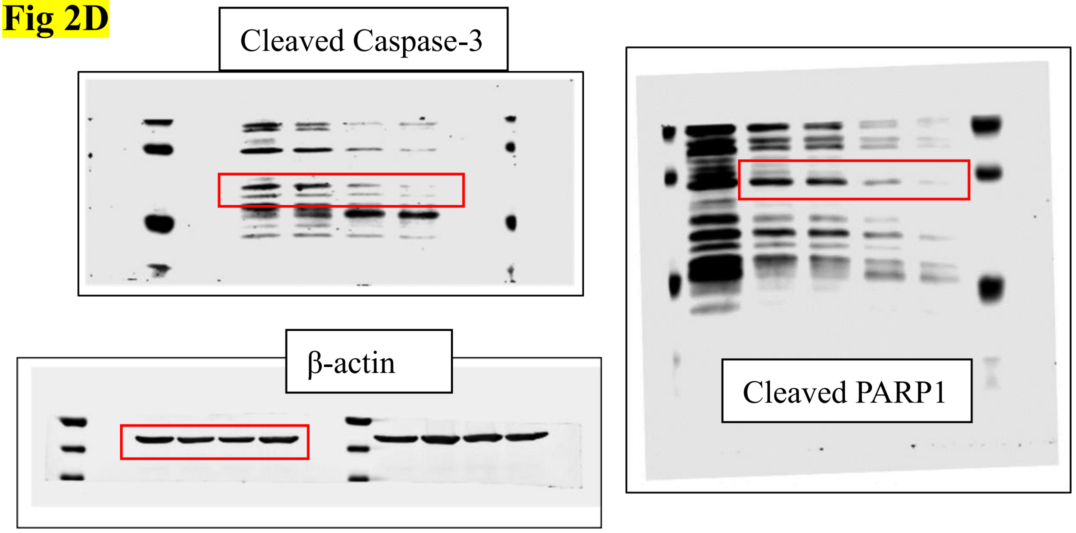

Fig 2E

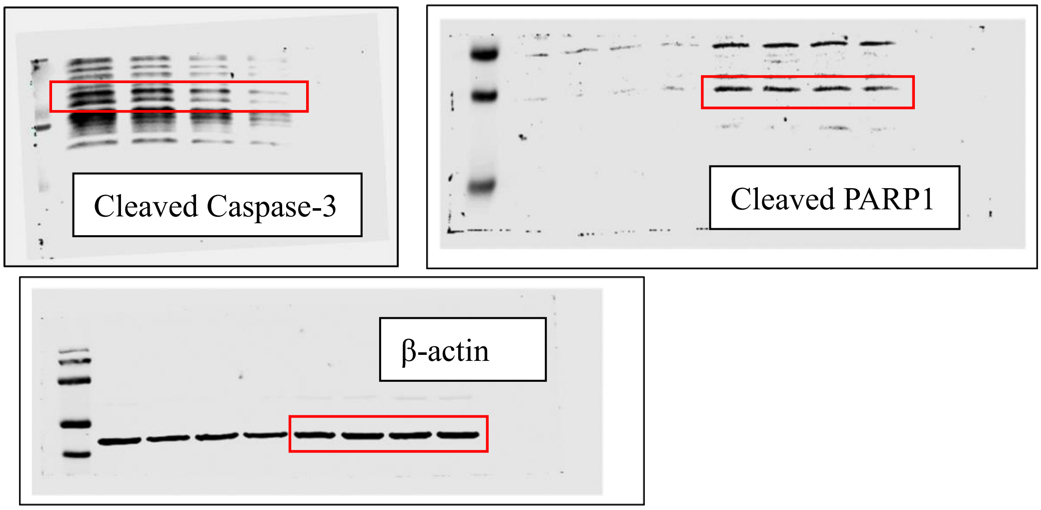

Fig 2F

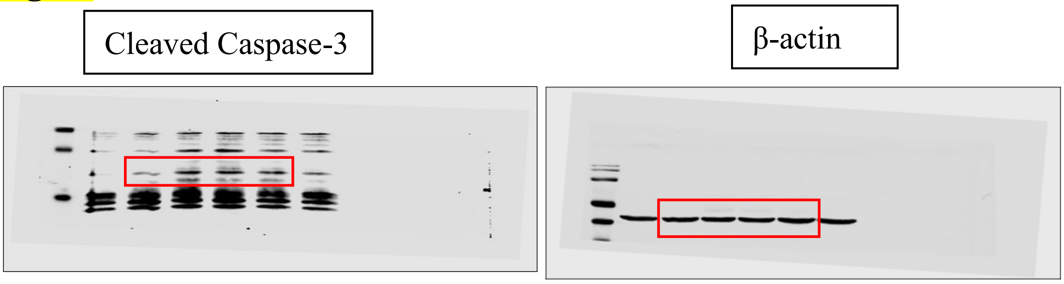

Fig 2G

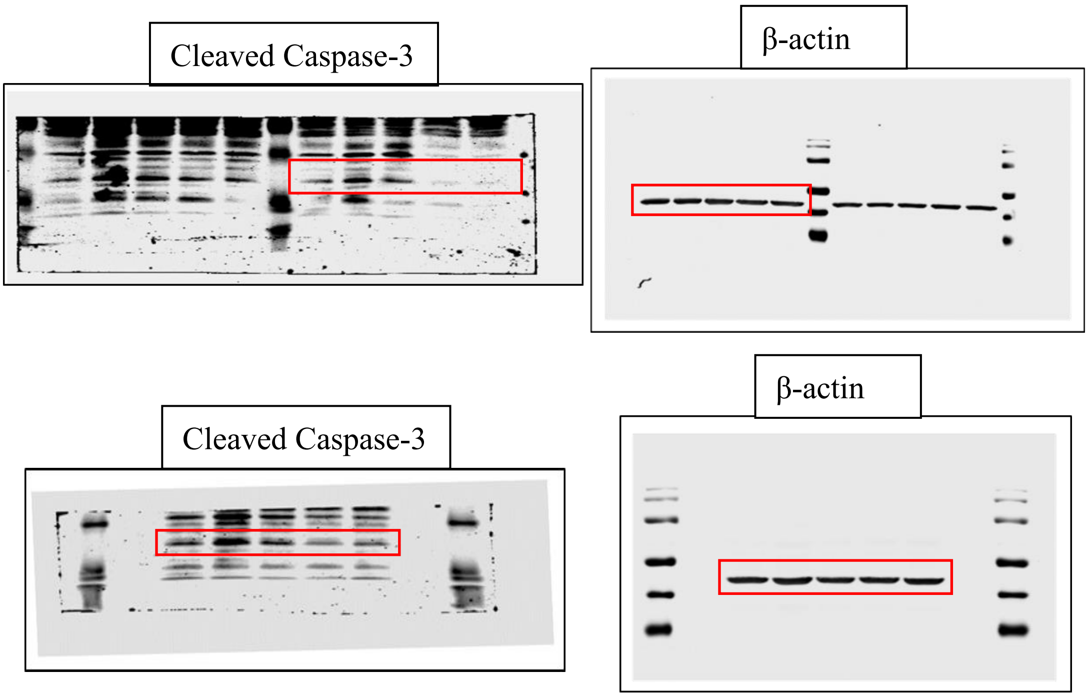

Fig 3A

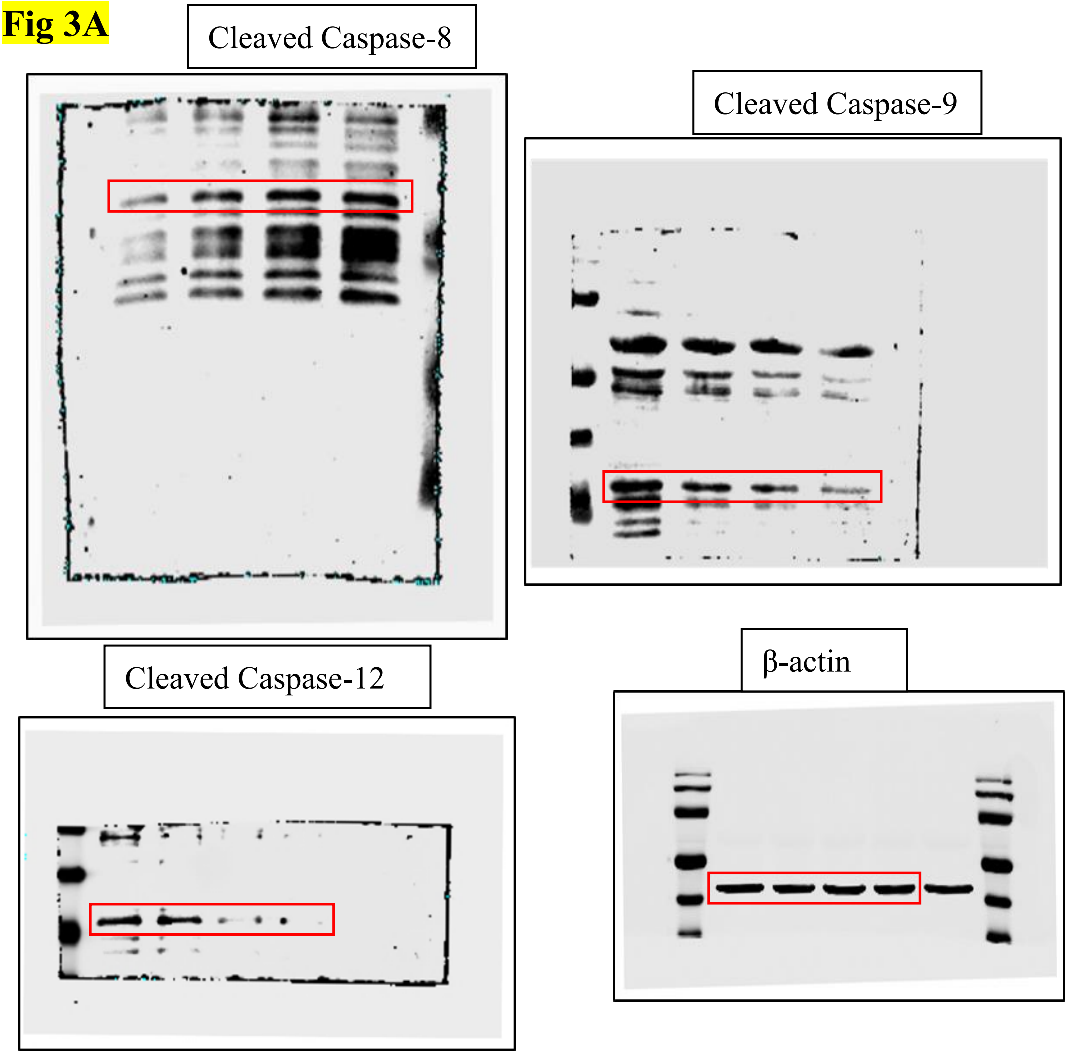
Cleaved Caspase-8
Cleaved Caspase-9
Cleaved Caspase-12
β-actin

Fig 3B

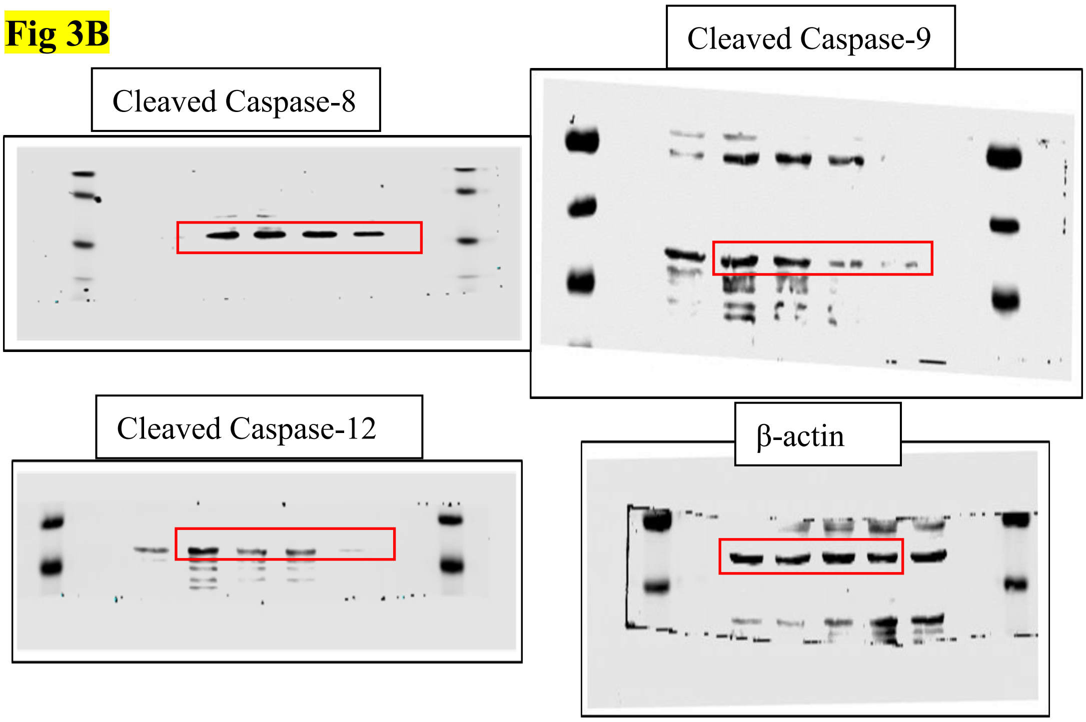
Cleaved Caspase-9
Cleaved Caspase-8
Cleaved Caspase-12
β-actin

Fig 3D

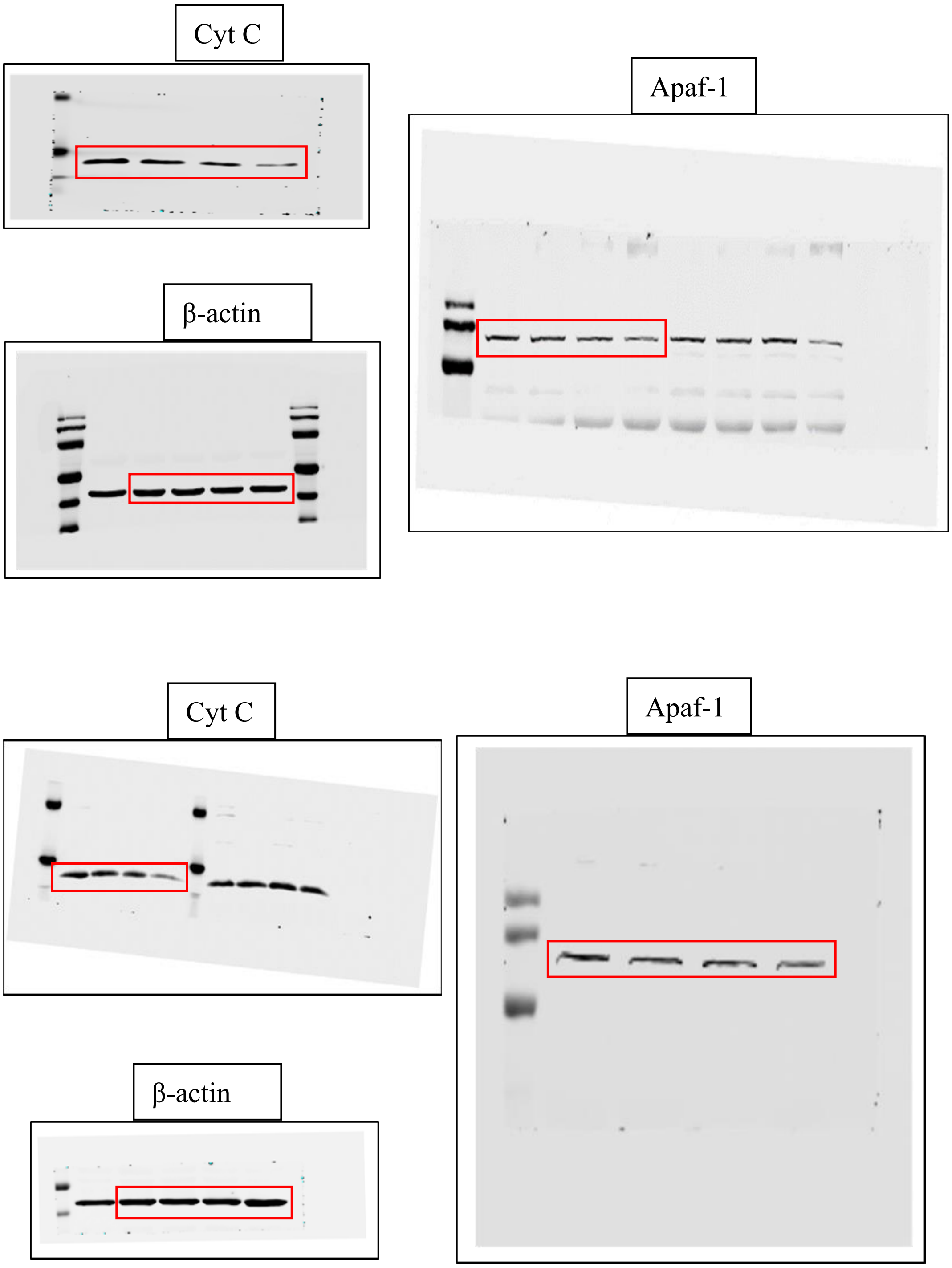

Fig 3E

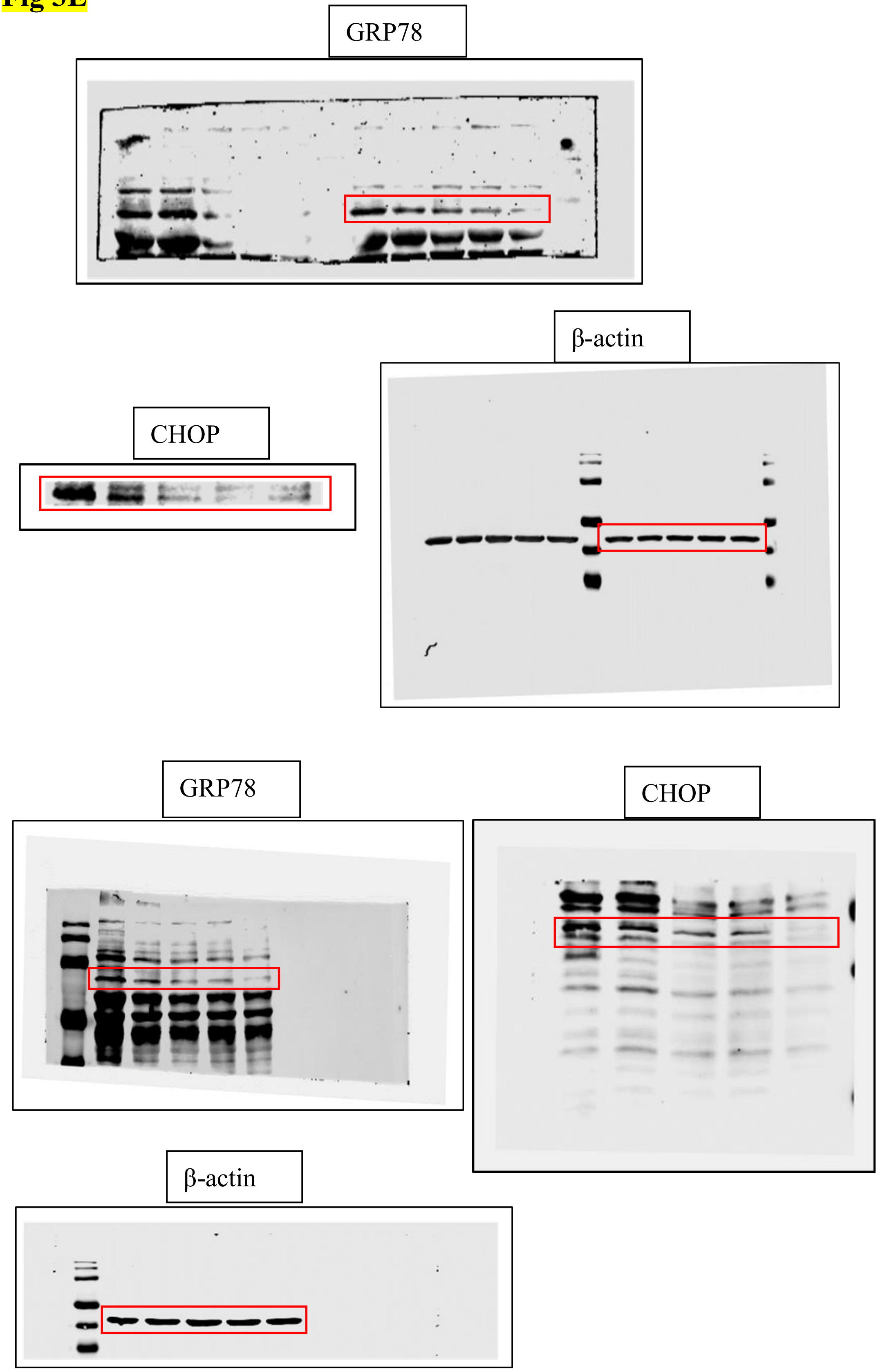

Fig 3G

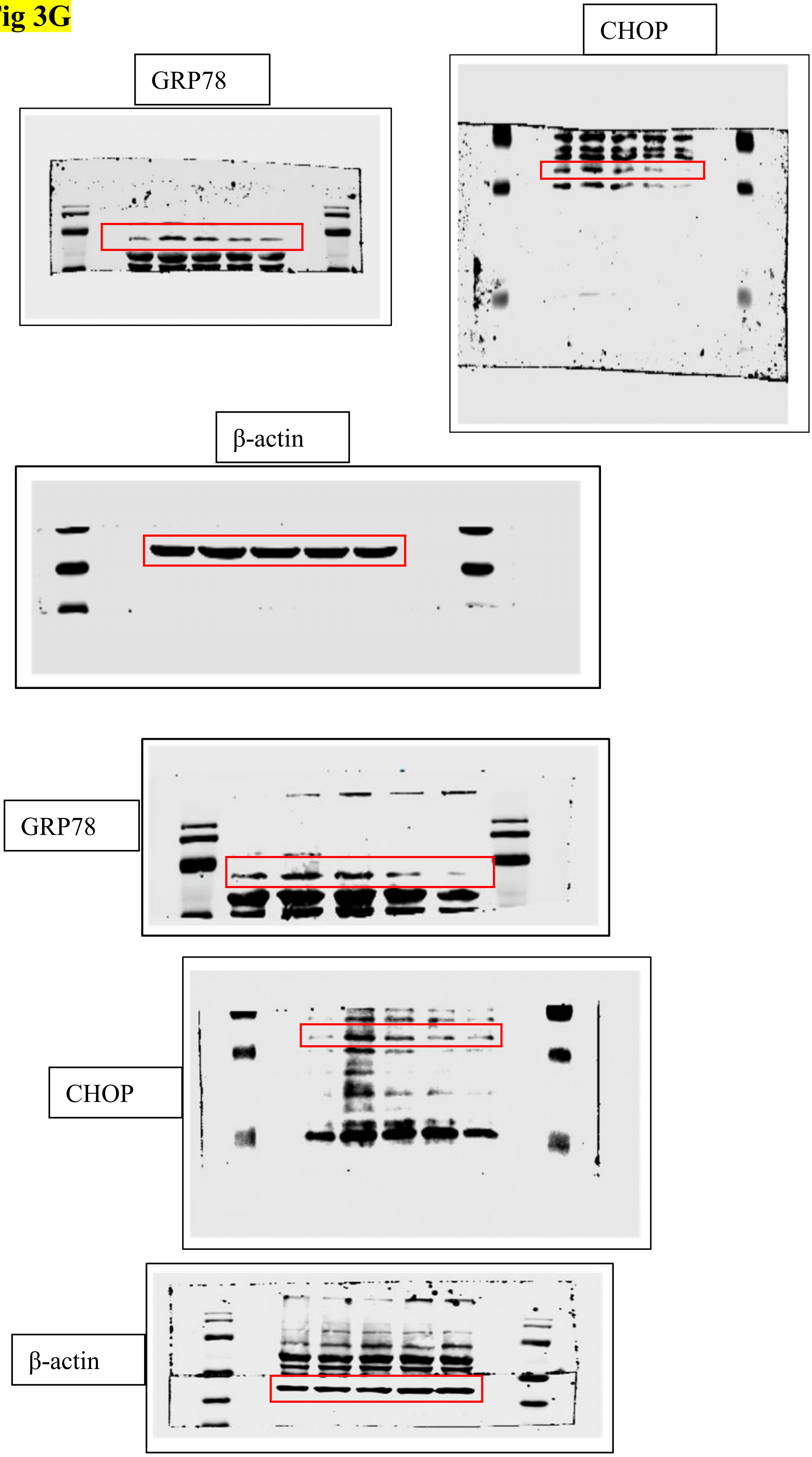

Fig 4B

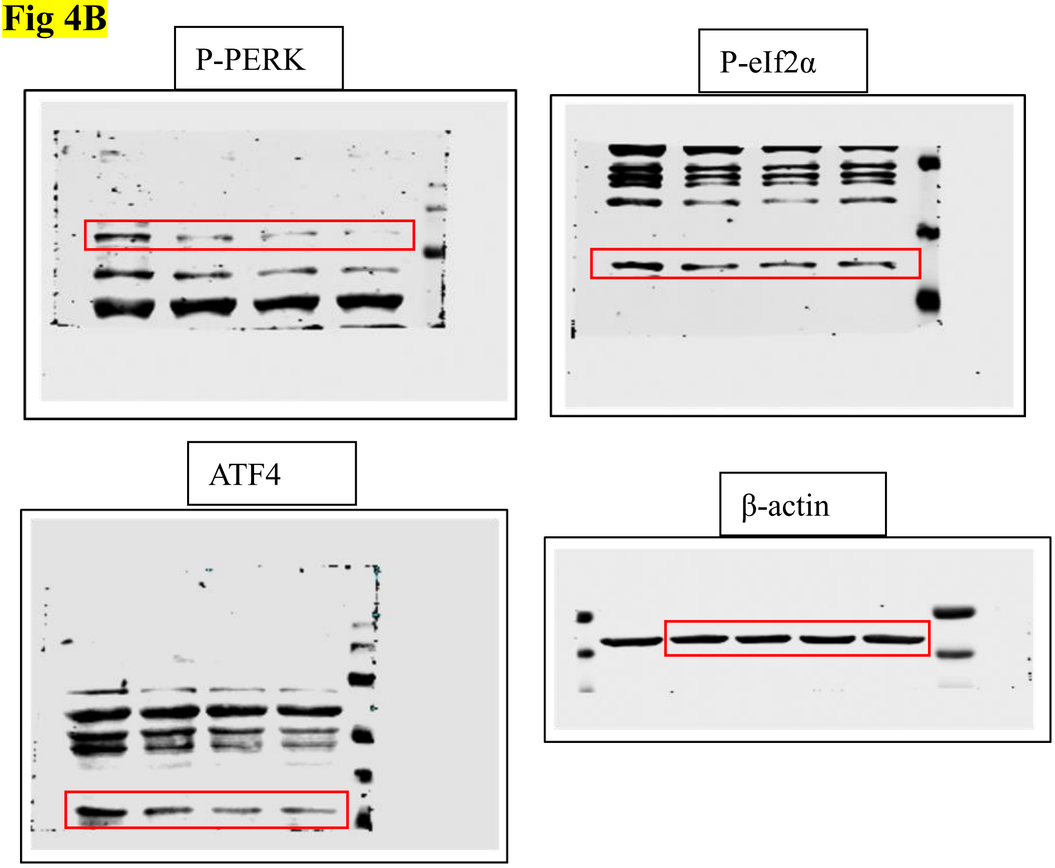

Fig 4C

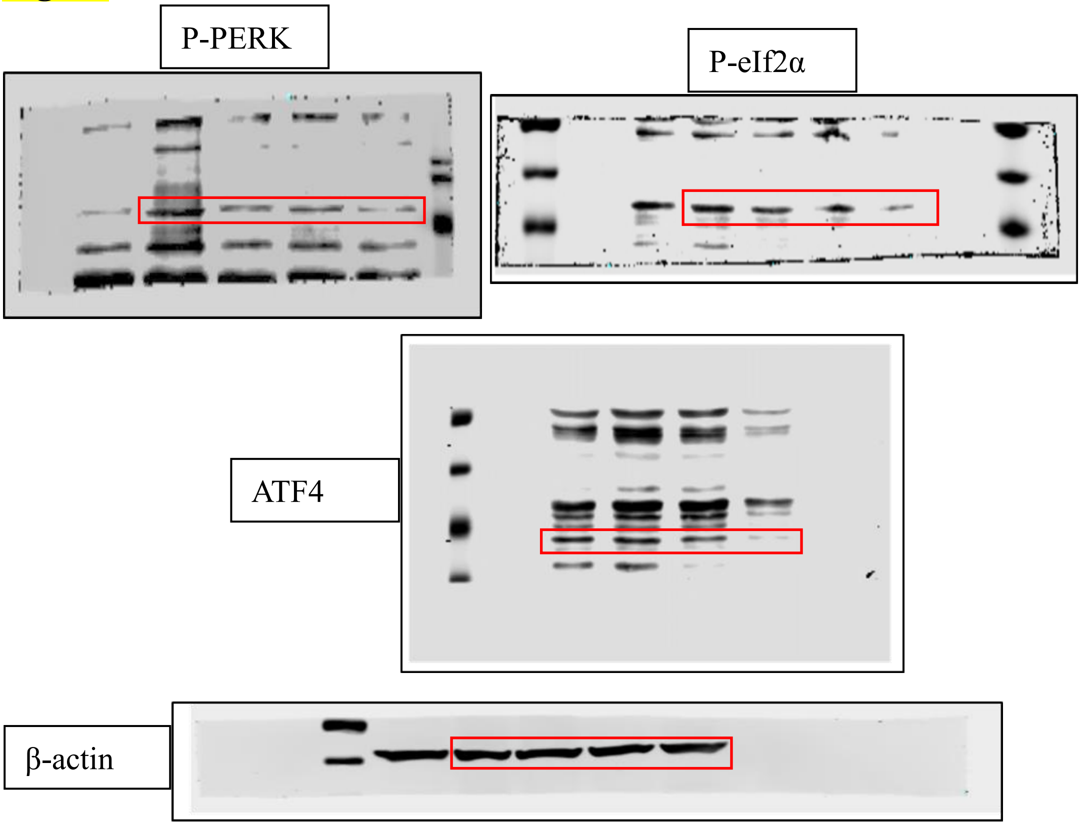

Fig 4D

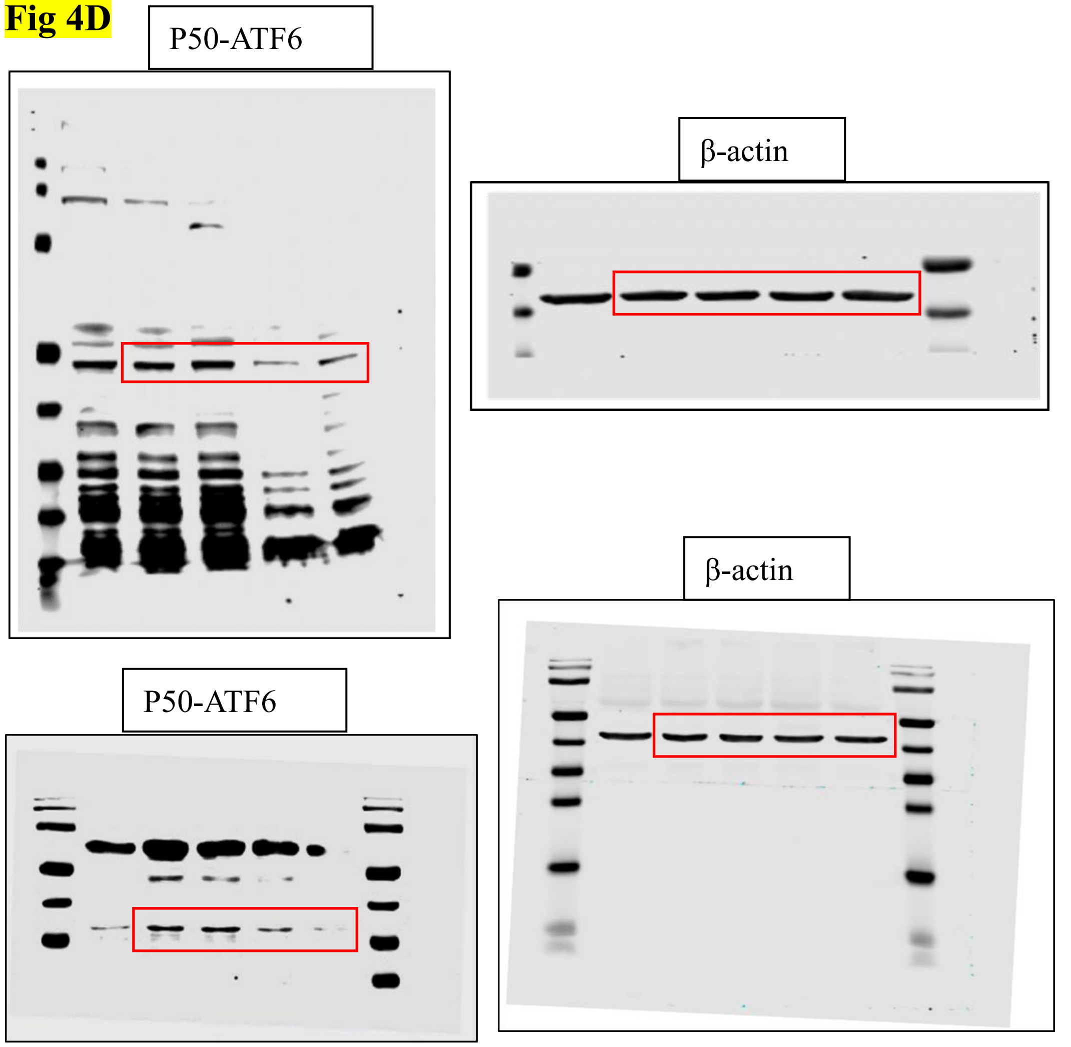

Fig 4E

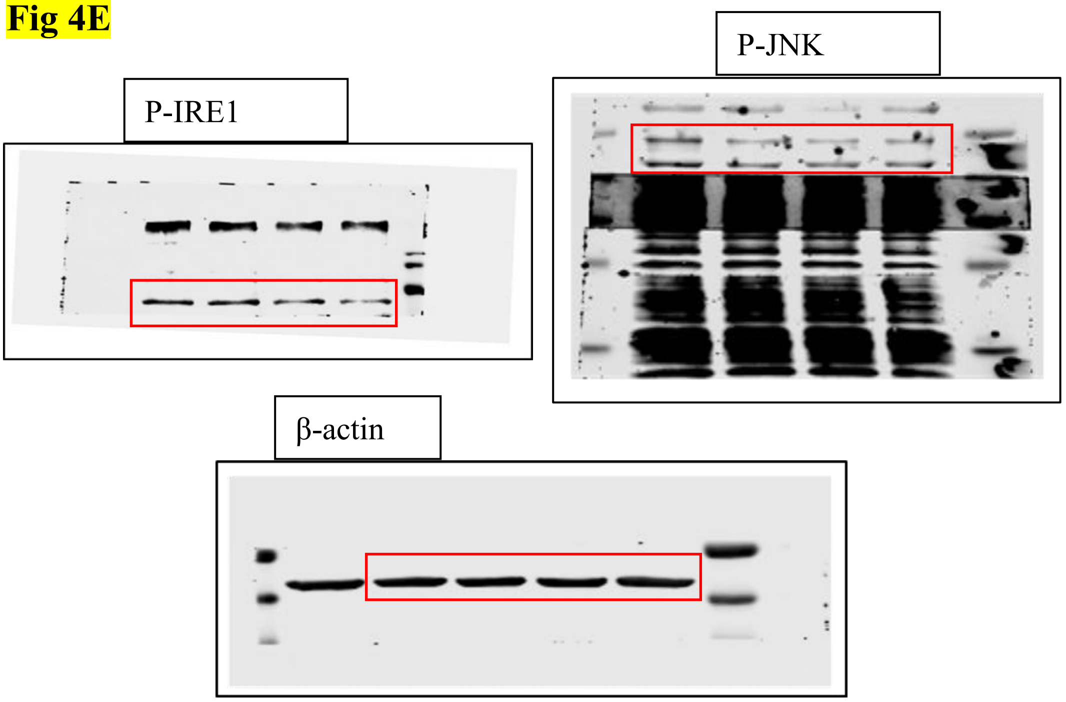

Fig 4F

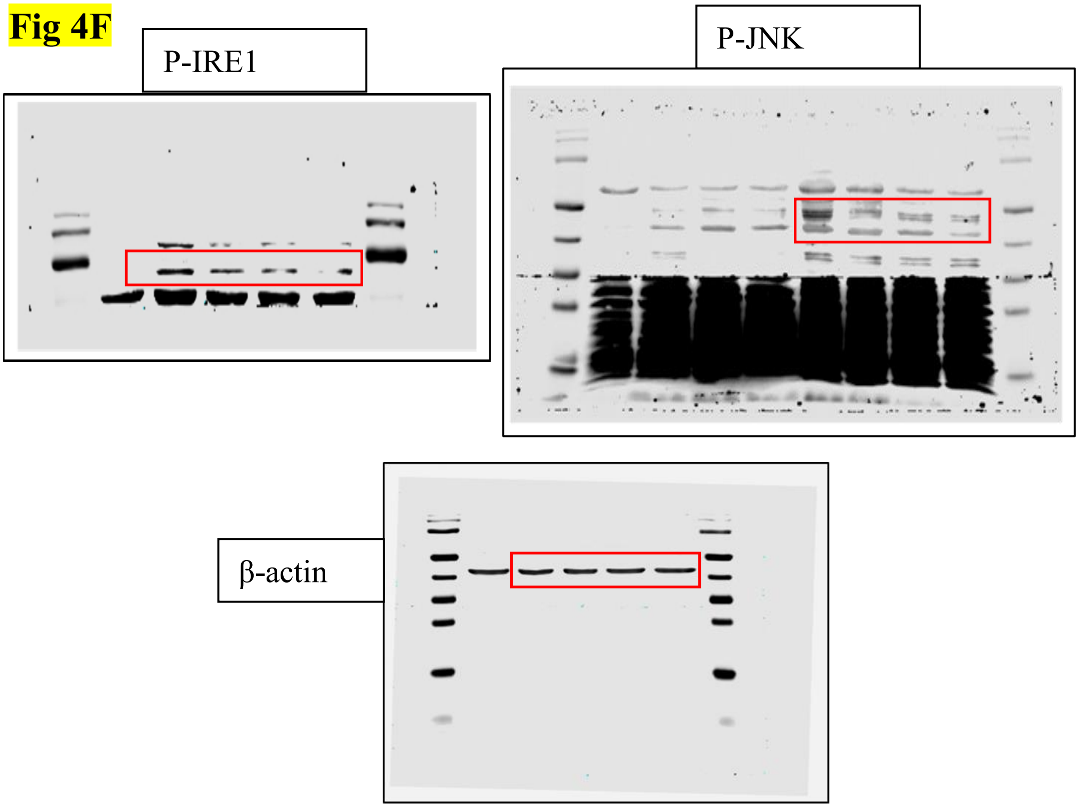

Fig 4H

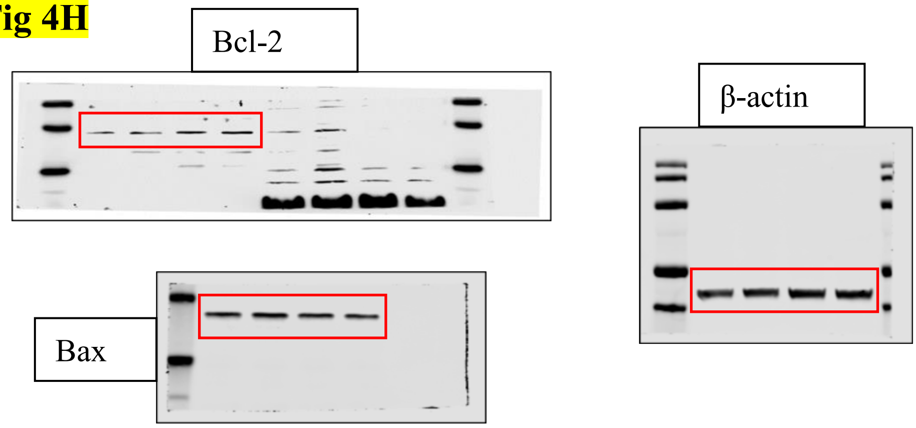

Fig 4I

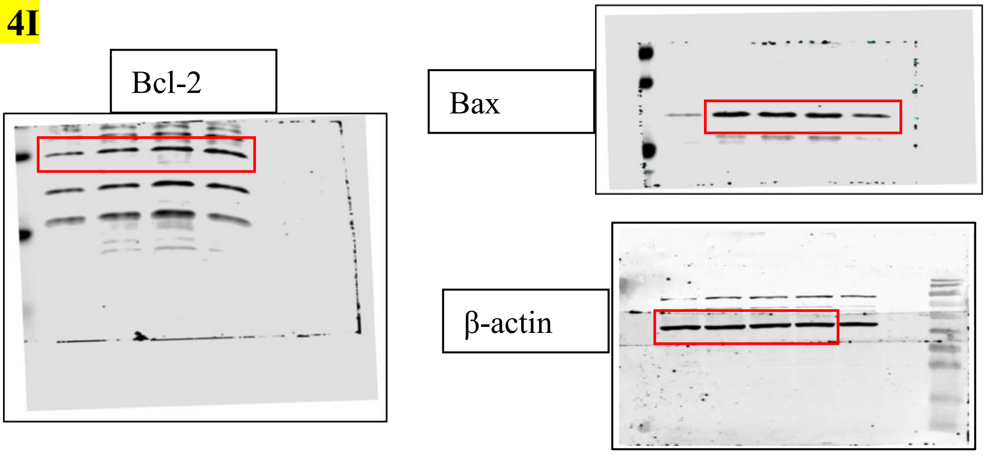

Fig 4J

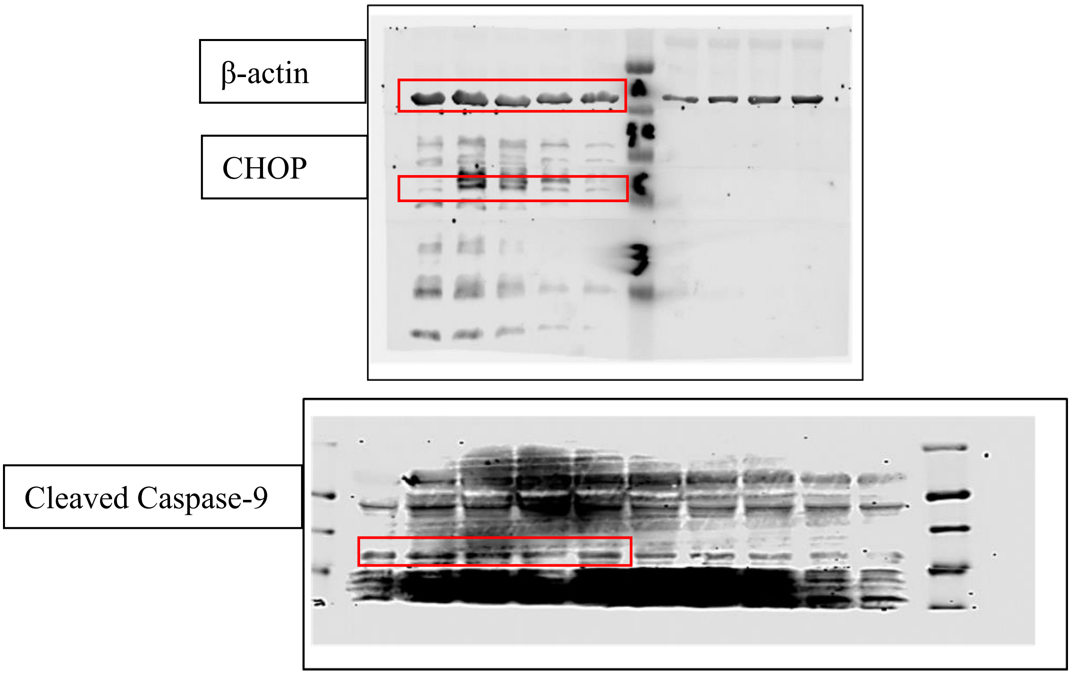

β-actin
CHOP
Cleaved Caspase-9

Fig 4K

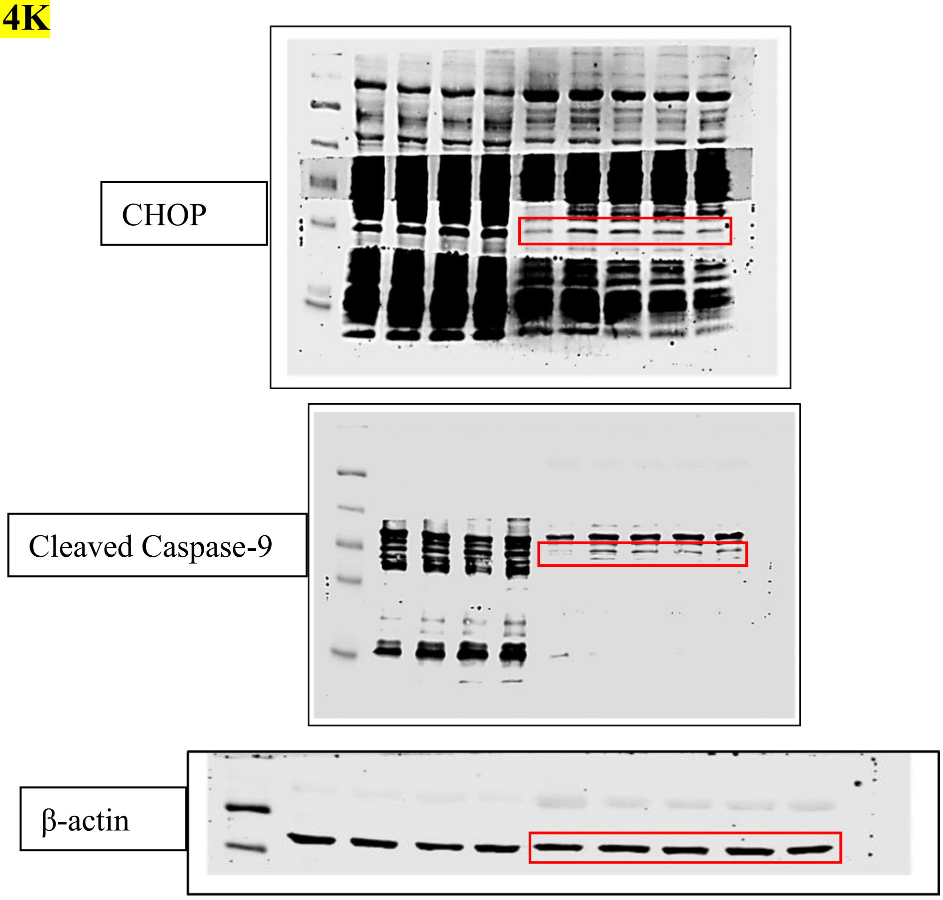

CHOP
Cleaved Caspase-9
β-actin

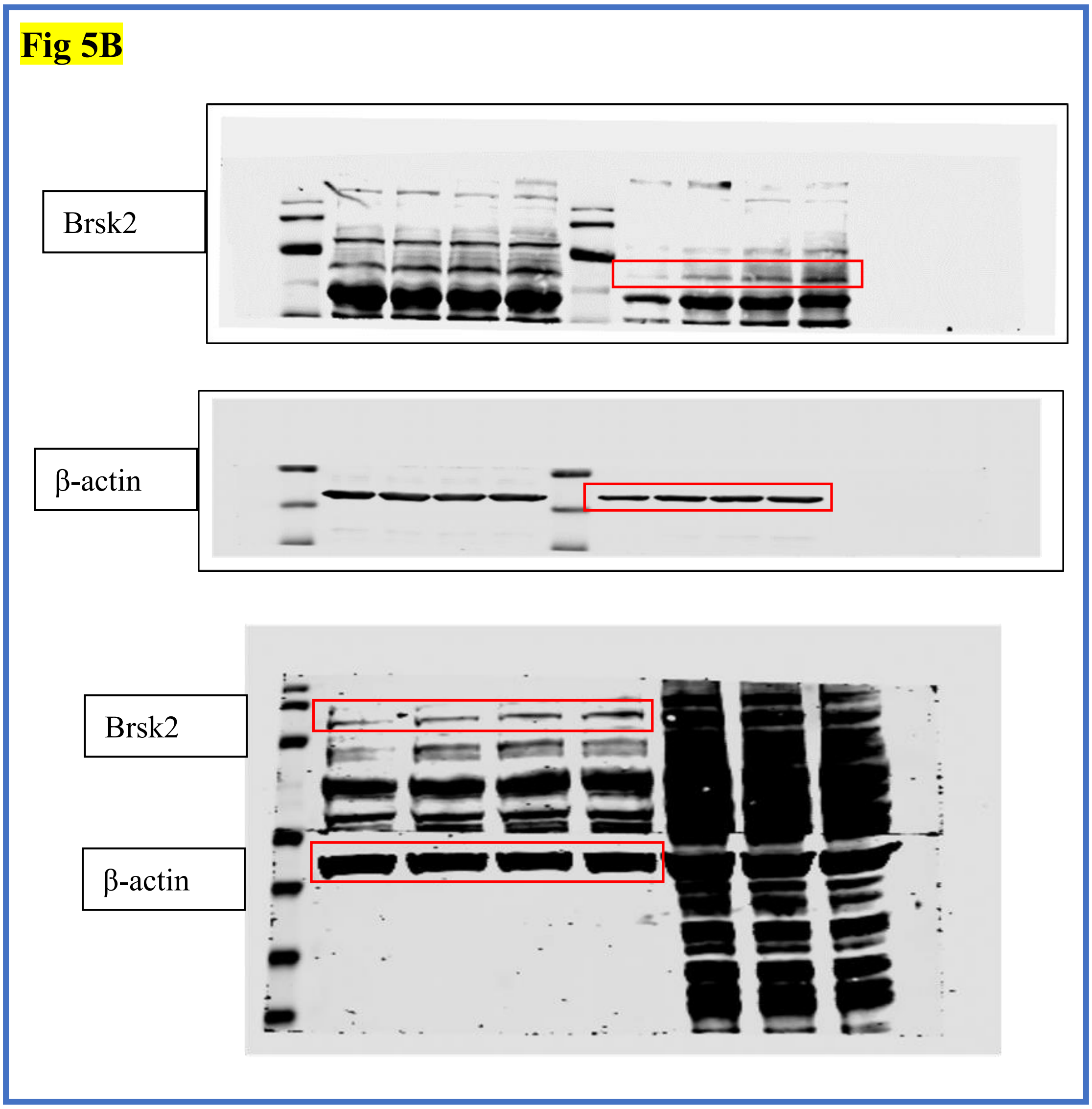
Fig 5B
Brsk2
β-actin
Brsk2
β-actin

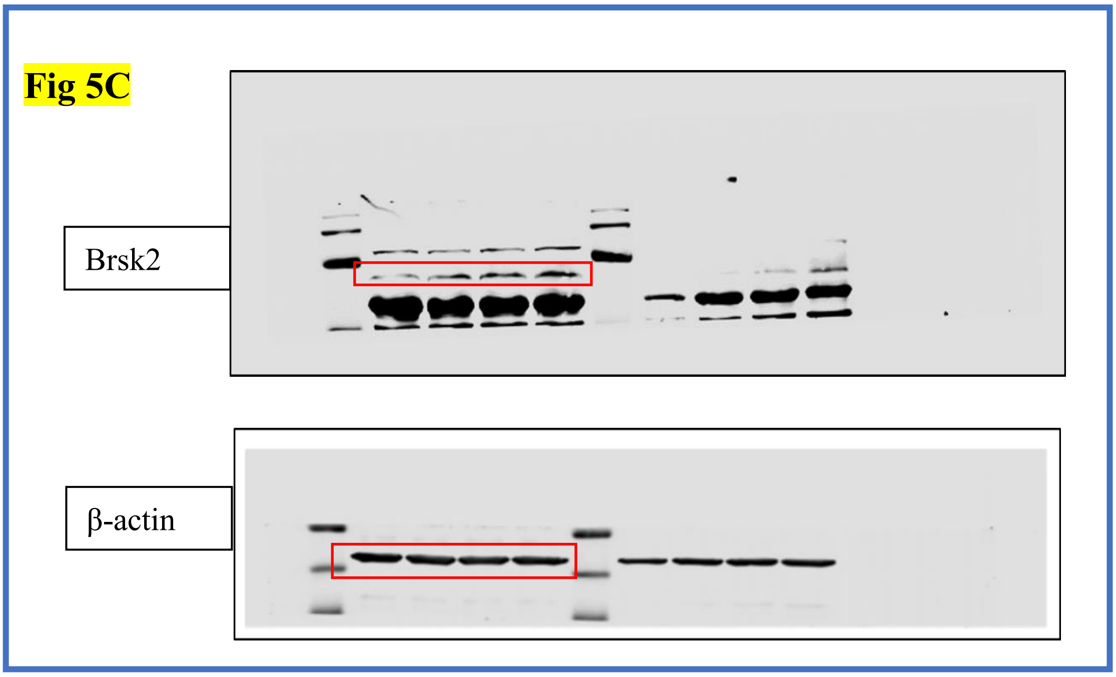
Fig 5C
Brsk2
β-actin

Fig 5D

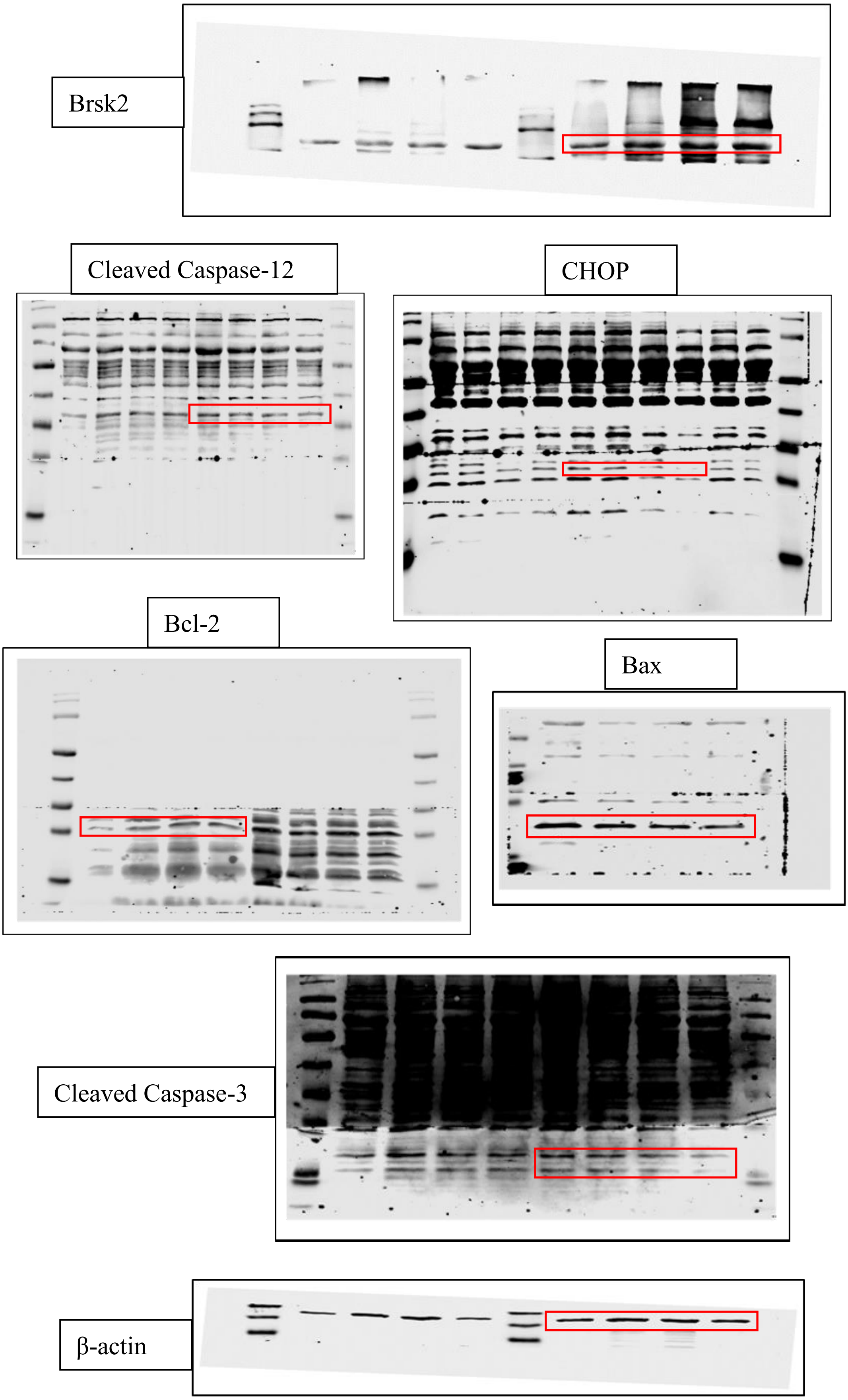
Brsk2
Cleaved Caspase-12
CHOP
Bcl-2
Bax
Cleaved Caspase-3
β-actin

Fig 5E

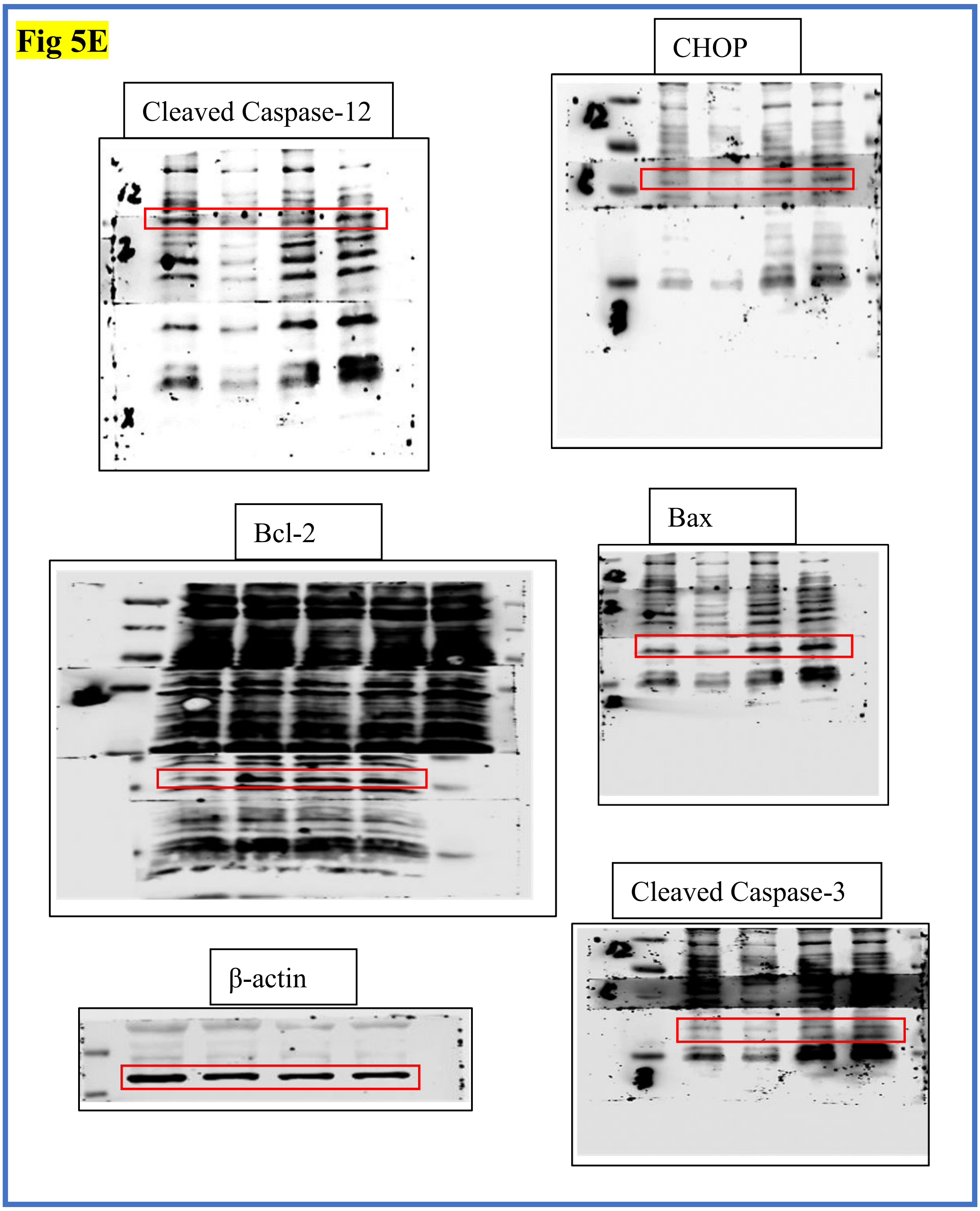
Cleaved Caspase-12
CHOP
Bcl-2
Bax
β-actin
Cleaved Caspase-3

Fig 5F

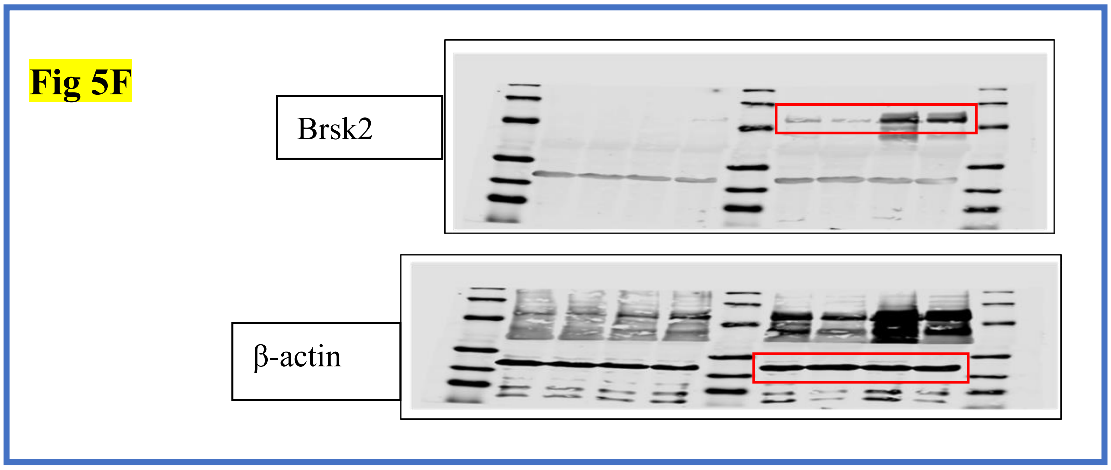
Brsk2
β-actin

Fig 5G

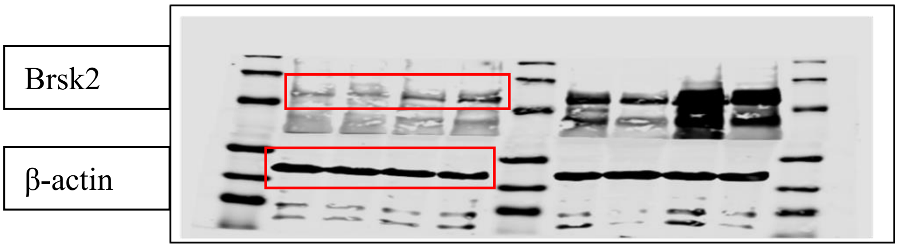

Fig 6A

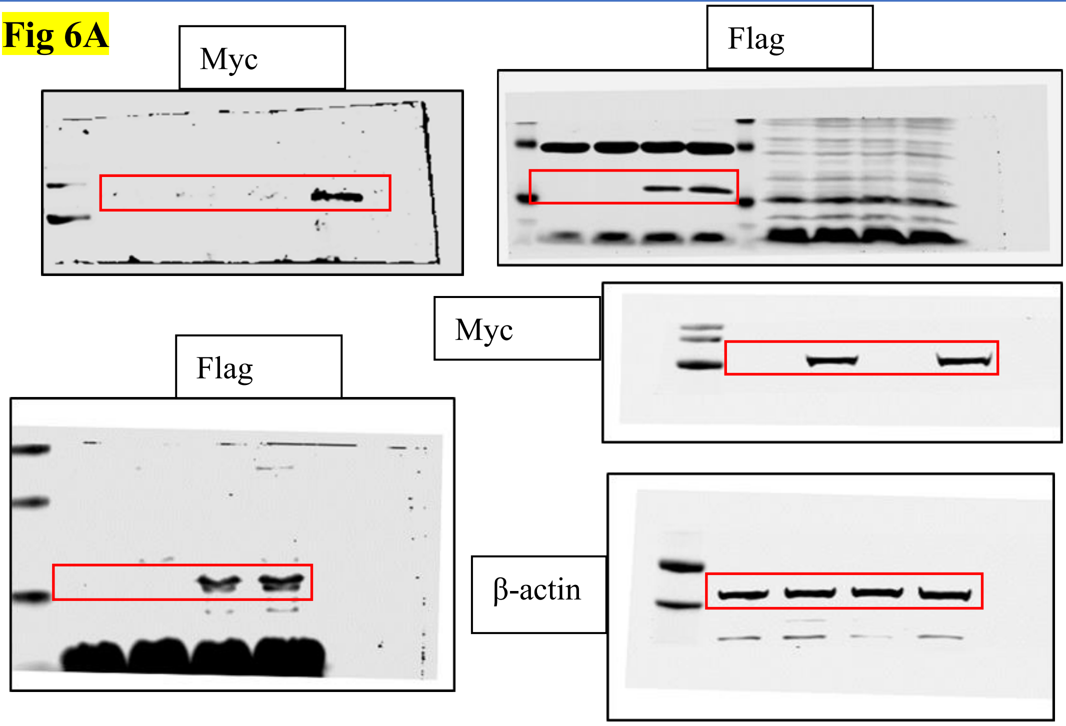

Fig 6B

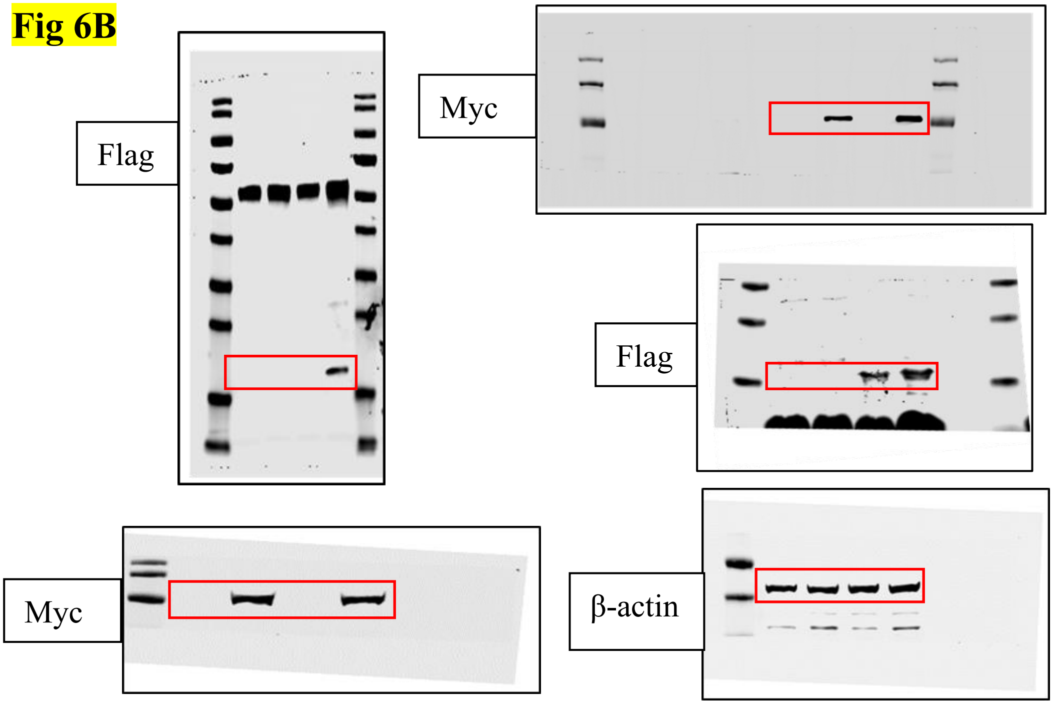

Fig 6D

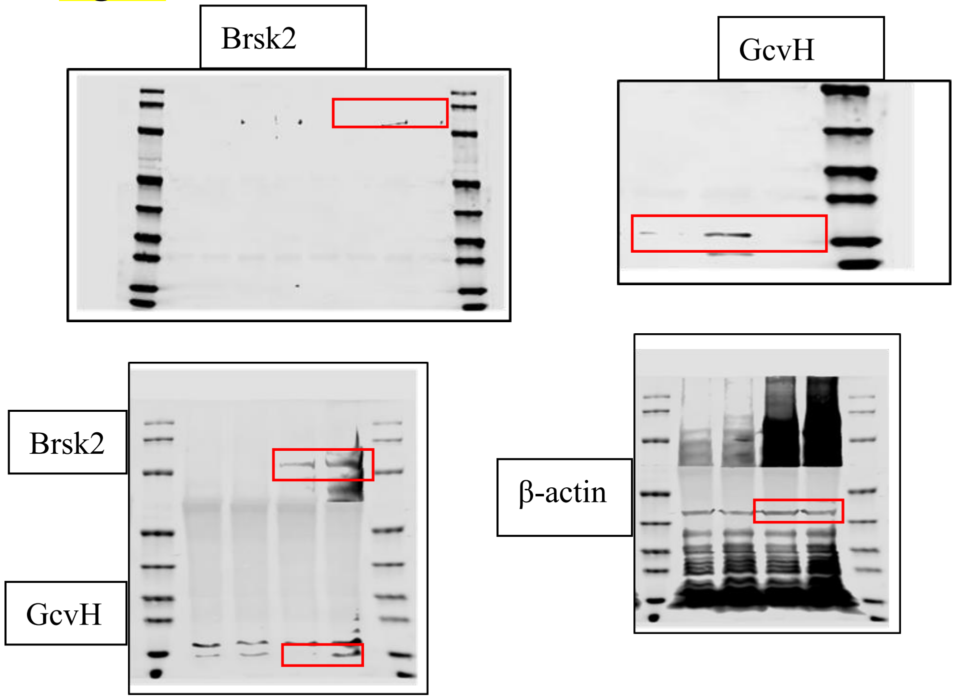

Fig 6E

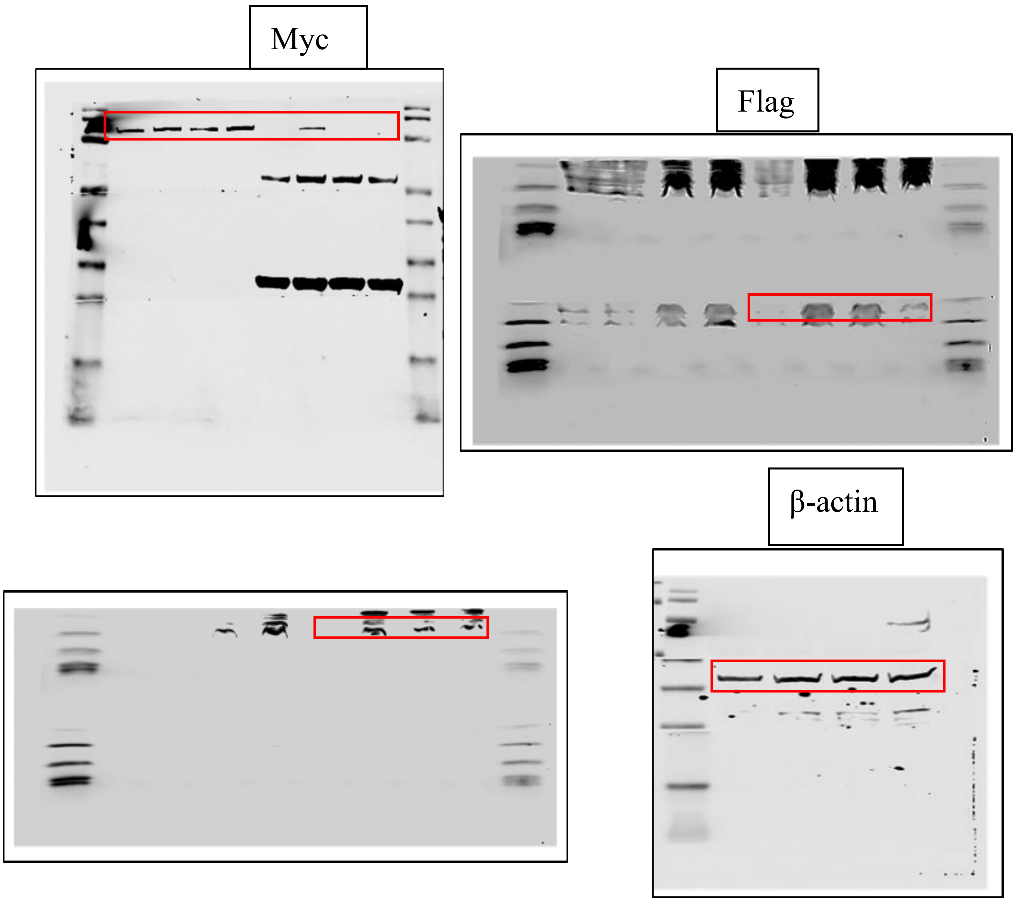

Fig 6F

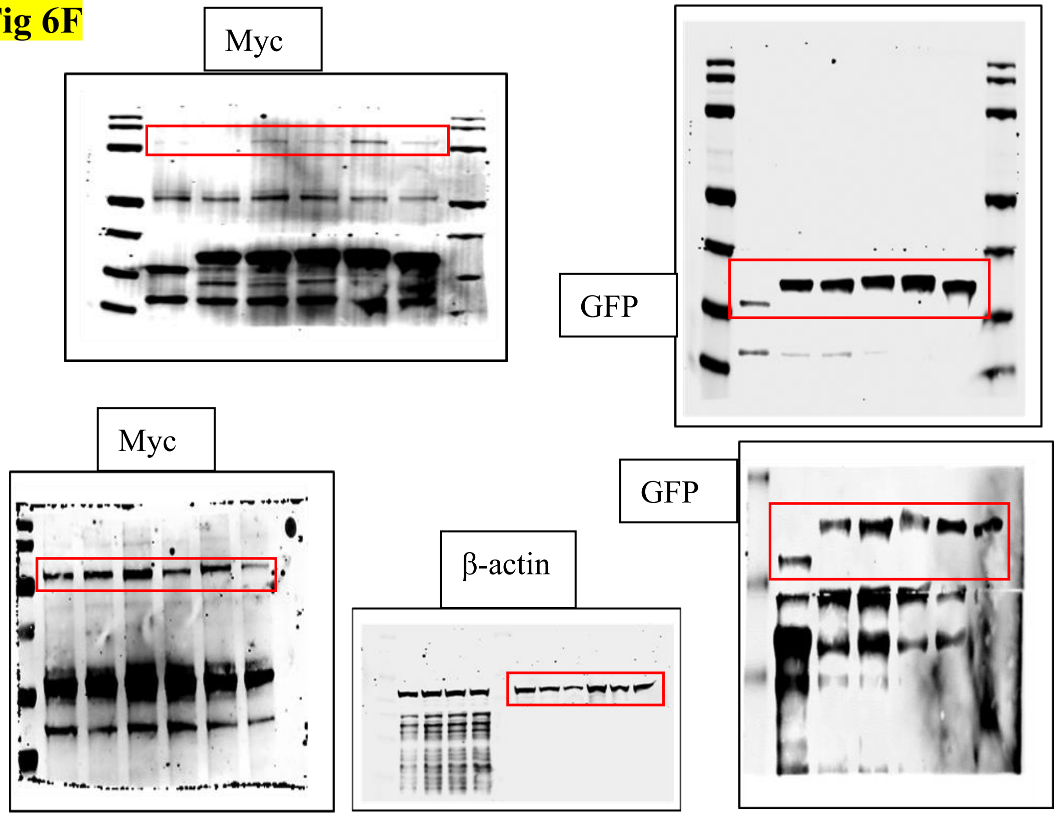

Fig 6G

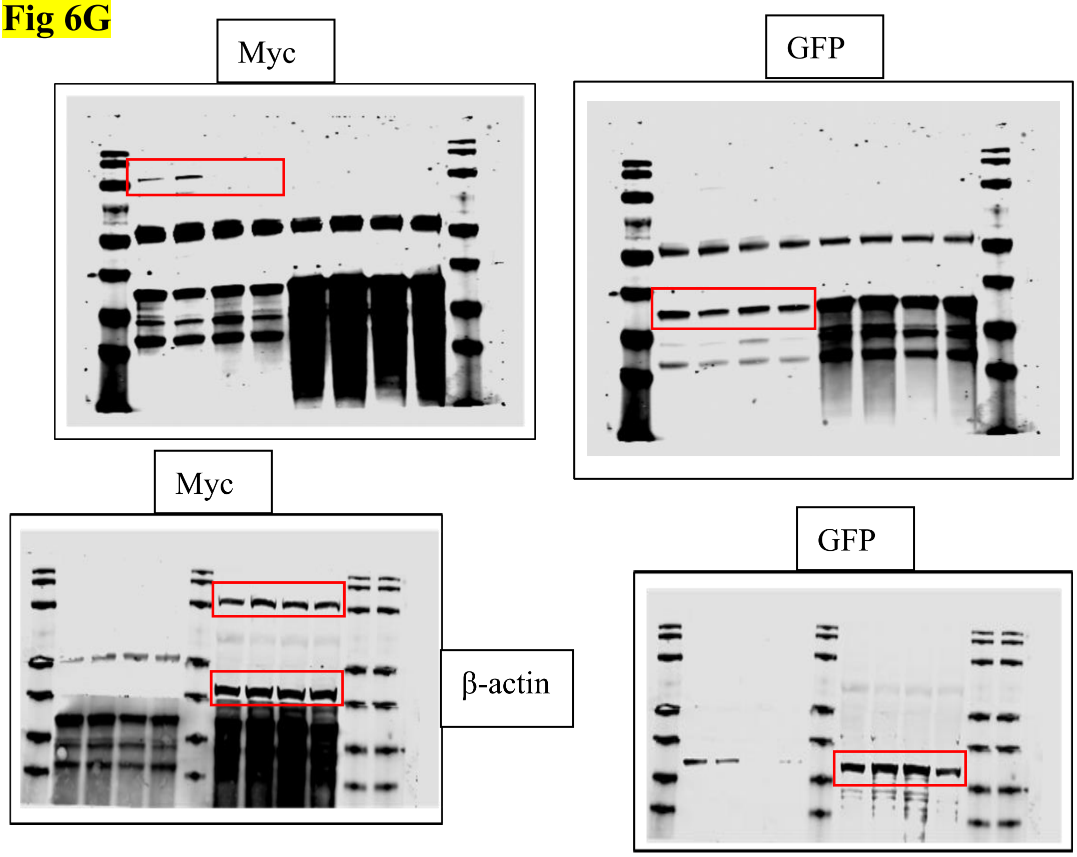

Fig 7A

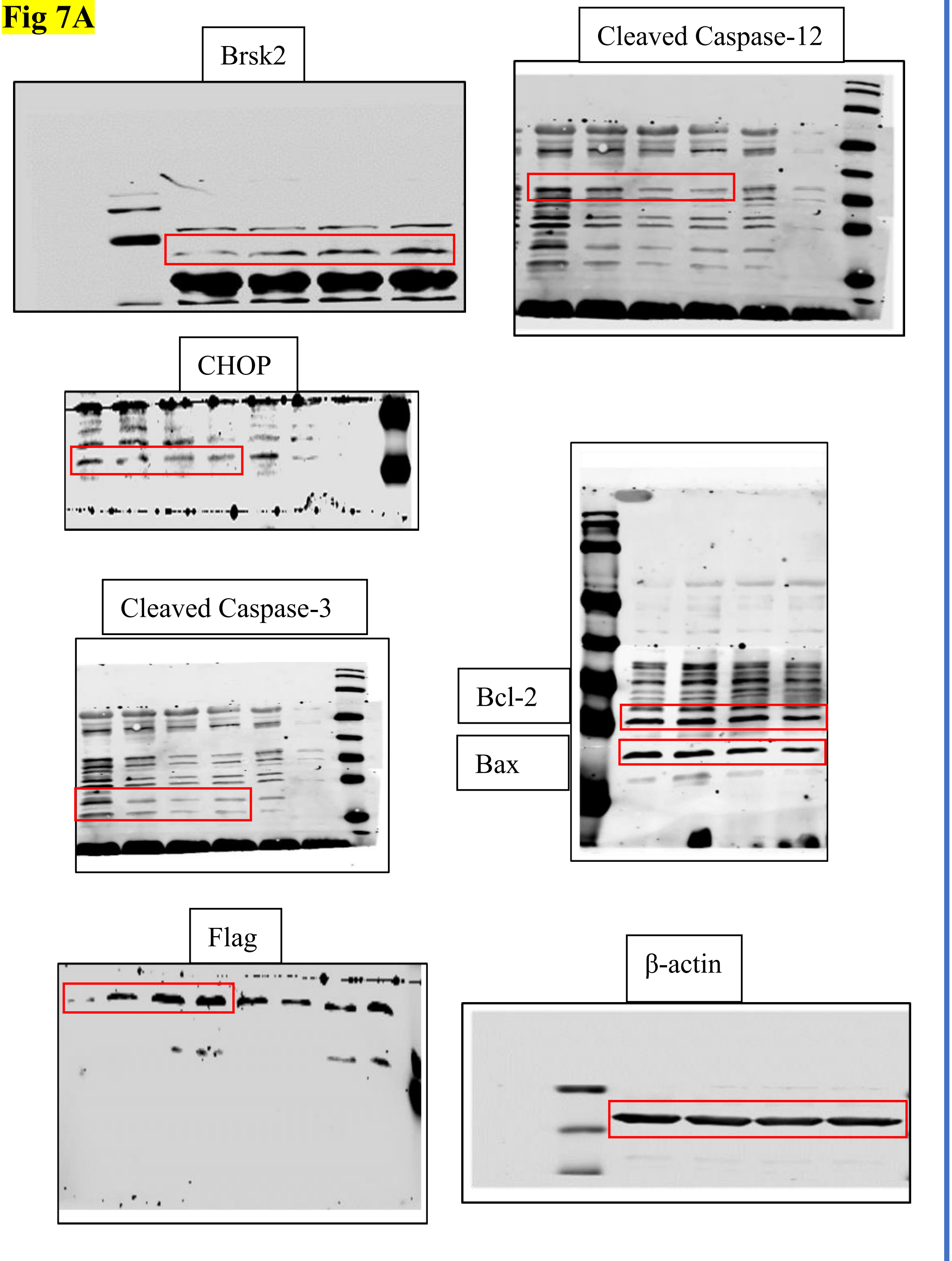
Brsk2
Cleaved Caspase-12
CHOP
Cleaved Caspase-3
Bcl-2
Bax
Flag
β-actin

Fig 7B

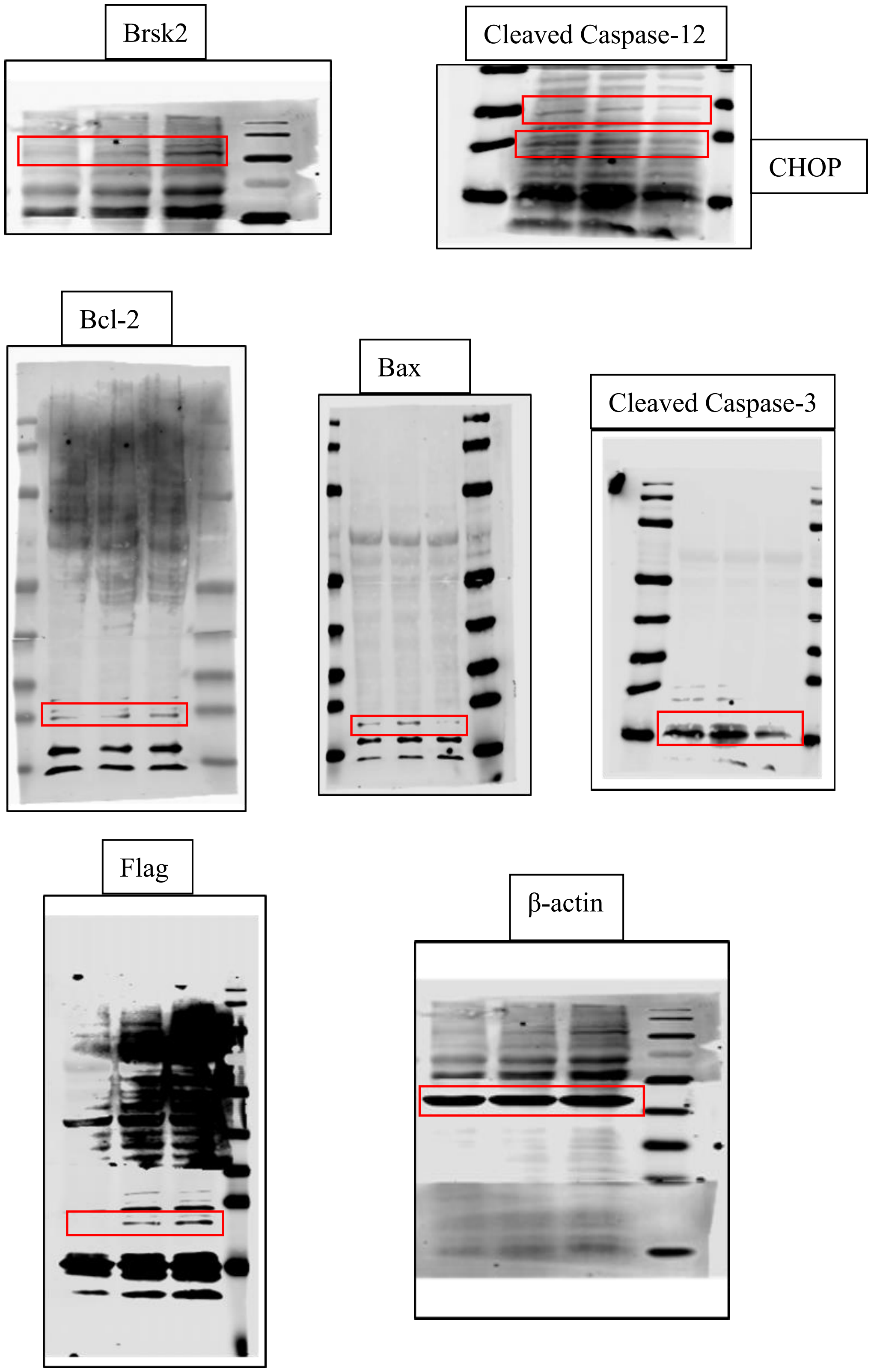

Brsk2
Cleaved Caspase-12
CHOP
Bcl-2
Bax
Cleaved Caspase-3
Flag
β-actin

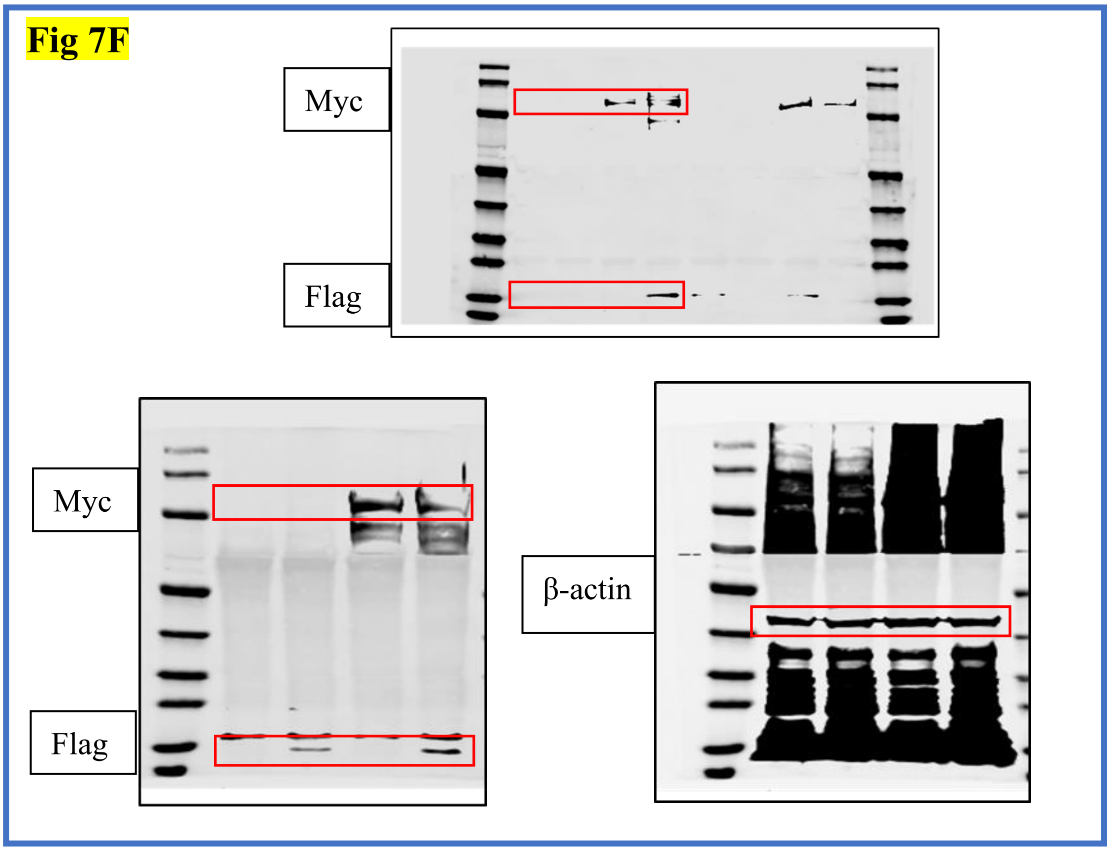
Fig 7F
Myc
Flag
Myc
Flag
β-actin

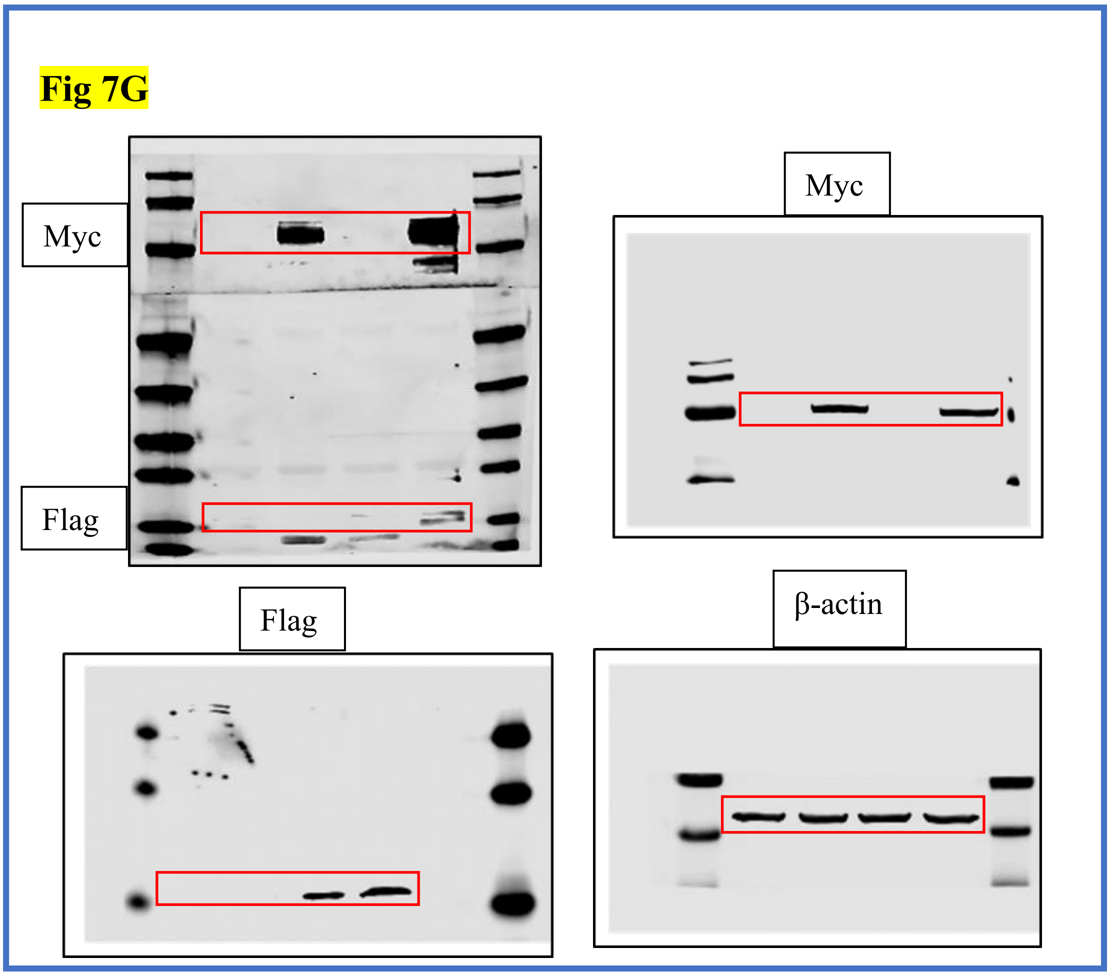
Fig 7G
Myc
Flag
Myc
Flag
β-actin

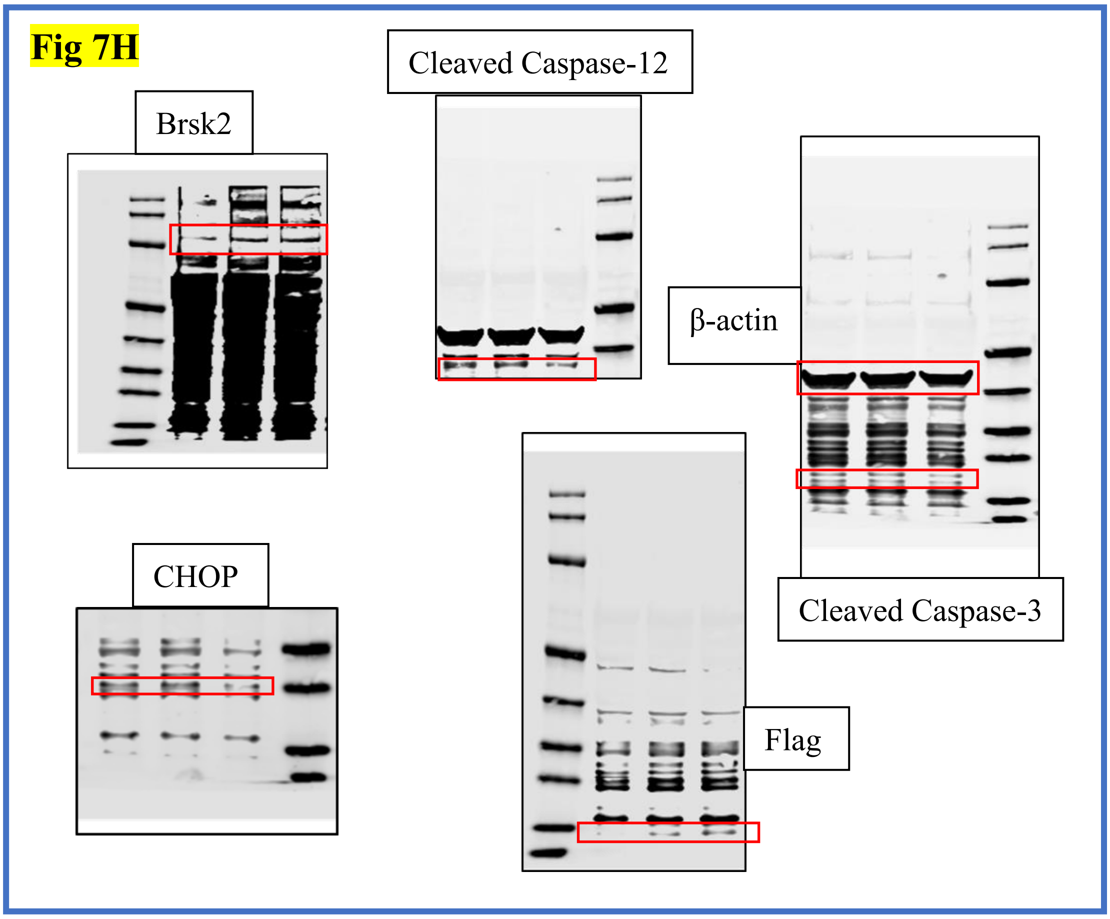
Fig 7H
Brsk2
Cleaved Caspase-12
β-actin
Cleaved Caspase-3
CHOP
Flag

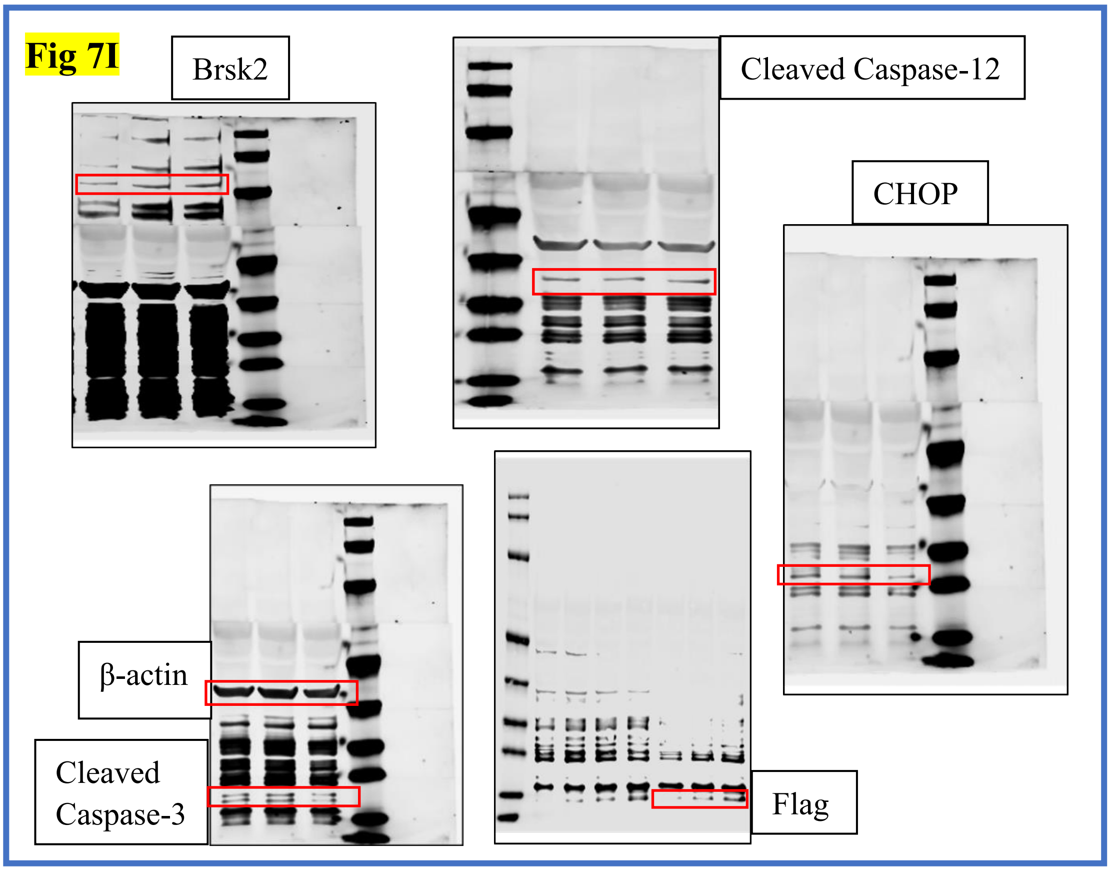
Fig 7I
Brsk2
Cleaved Caspase-12
CHOP
β-actin
Cleaved Caspase-3
Flag

Fig S1C

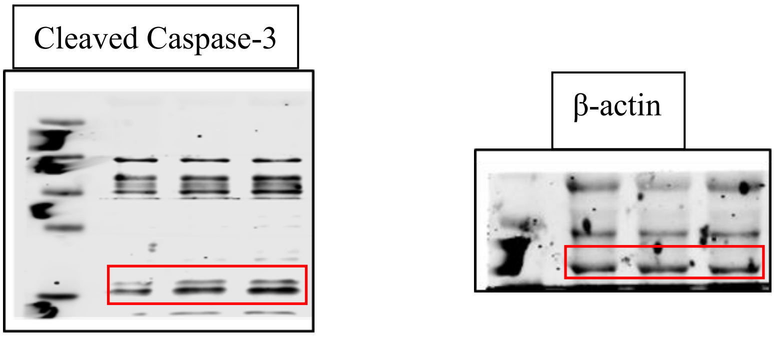

Fig S4B

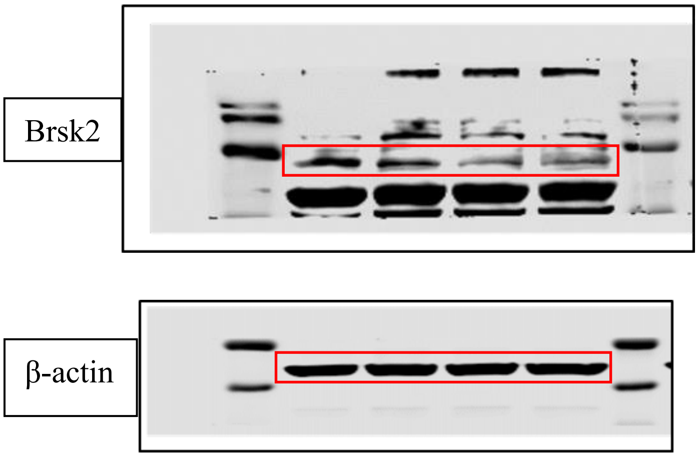

Fig S4D

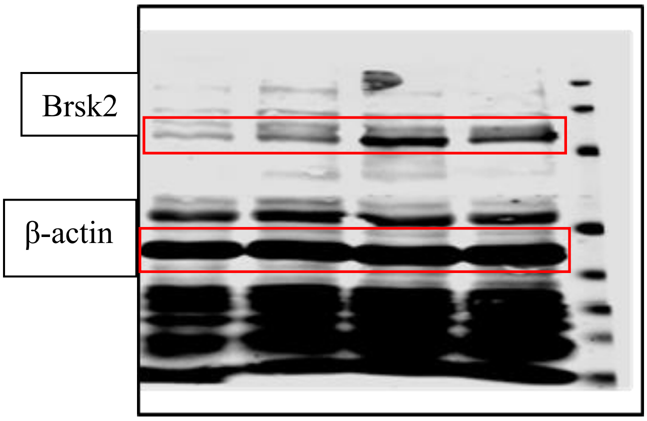

Supplement: S2 Data — (PDF) [file ppat.1012266.s008.pdf]
